# Autoantibody profiles associated with clinical features in psychotic disorders

**DOI:** 10.1038/s41398-021-01596-0

**Published:** 2021-09-13

**Authors:** August Jernbom Falk, Cherrie Galletly, David Just, Catherine Toben, Bernhard T. Baune, Scott R. Clark, Dennis Liu, Peter Nilsson, Anna Månberg, K. Oliver Schubert

**Affiliations:** 1 Department of Protein Science, KTH Royal Institute of Technology, SciLifeLab, Stockholm, Sweden; 2 Discipline of Psychiatry, Adelaide Medical School, University of Adelaide, Adelaide, SA Australia; 3 Northern Adelaide Mental Health Services, SA Health, Adelaide, SA Australia; 4 Ramsay Health Care (SA) Mental Health, The Adelaide Clinic, Adelaide, SA Australia; 5 Department of Mental Health, University of Münster, Münster, Germany; 6 Lab Division of Molecular Neurobiology of Mental Health, University of Münster, Münster, Germany; 7 Department of Psychiatry, Melbourne Medical School, The University of Melbourne, Melbourne, VIC Australia; 8 The Florey Institute of Neuroscience and Mental Health, The University of Melbourne, Parkville, VIC Australia; 9 Department of Psychiatry, SUNY Upstate Medical University, Syracuse, NY USA; 10 School of Psychiatry, University of New South Wales, Sydney, NSW Australia

**Keywords:** Diagnostic markers, Molecular neuroscience, Psychiatric disorders

## Abstract

Autoimmune processes are suspected to play a role in the pathophysiology of psychotic disorders. Better understanding of the associations between auto-immunoglobulin G (IgG) repertoires and clinical features of mental illness could yield novel models of the pathophysiology of psychosis, and markers for biological patient stratification. We undertook cross-sectional detection and quantification of auto-IgGs in peripheral blood plasma of 461 people (39% females) with established psychotic disorder diagnoses. Broad screening of 24 individuals was carried out on group level in eight clinically defined groups using planar protein microarrays containing 42,100 human antigens representing 18,914 proteins. Autoantibodies indicated by broad screening and in the previous literature were measured using a 380-plex bead-based array for autoantibody profiling of all 461 individuals. Associations between autoantibody profiles and dichotomized clinical characteristics were assessed using a stepwise selection procedure. Broad screening and follow-up targeted analyses revealed highly individual autoantibody profiles. Females, and people with family histories of obesity or of psychiatric disorders other than schizophrenia had the highest overall autoantibody counts. People who had experienced subjective thought disorder and/or were treated with clozapine (trend) had the lowest overall counts. Furthermore, six autoantibodies were associated with specific psychopathology symptoms: anti-AP3B2 (persecutory delusions), anti-TDO2 (hallucinations), anti-CRYGN (initial insomnia); anti-APMAP (poor appetite), anti-OLFM1 (above-median cognitive function), and anti-WHAMMP3 (anhedonia and dysphoria). Future studies should clarify whether there are causal biological relationships, and whether autoantibodies could be used as clinical markers to inform diagnostic patient stratification and choice of treatment.

## Introduction

Mental illnesses with psychosis as a clinical feature such as schizophrenia (SCZ), schizoaffective disorder (SCZAD), bipolar disorder (BD), and psychotic major depressive disorder affect about 3% of the population [1] and are associated with long-term disability and catastrophic outcomes such as suicide. Available treatments are of modest overall efficacy [2], and there are no proven prevention strategies [3]. A better understanding of the underlying molecular mechanisms that drive disorder risk and progression is required to improve clinical outcomes.

Immune system abnormalities have been found across a range of psychiatric disorders [4] and findings are underpinned by findings of large scale genetic- [5], gene expression- [6], and proteomic [7] studies. Autoimmunity, characterized by the production of antibodies against the body’s own antigens, is a feature of immune system dysfunction and could play a role in mental disorder pathophysiology. For example, autoantibodies against neuroreceptors such as the N-methyl-D-aspartate receptor (NMDAR) are found in about 1–5% of various neuropsychiatric patient groups and healthy individuals, and in some cases are causally associated with psychopathology [8]. Further, meta-analytic epidemiological evidence demonstrates that non-neurological autoimmune disorders are more common in people with mental disorders than in the general population [9] and common auto-immunoglobulin G (IgG) proteins are increased in people with SCZ [10]. Another study found that SCZ patients had increased reactivity against autoantigens implicated in SCZ genome-wide association studies [11].

Recent advances in autoantibody profiling technologies facilitate more fine-grained analyses of the role of autoimmunity in medicine. Studies in healthy volunteers have shown that individuals possess a highly personalized repertoire of auto-IgGs that is stable over time and could be described as an “autoimmune fingerprint” [12]. Analyses of autoantibody repertoires in cohorts of people with psychiatric disorders could therefore identify novel disorder- and symptom-associated autoantibody signatures, in turn providing novel insights into pathophysiology and possible new treatments. Demonstrating the feasibility and potential clinical utility of this approach, a previous untargeted autoimmune profiling study comparing young people with first-episode psychosis and healthy controls identified a novel autoantibody target in the N-terminal portion of the p antigen (PAGE) protein group (PAGE2B/PAGE2/PAGE5), which was found in cases only and associated with the later development of SCZ as opposed to other first-episode psychosis outcomes [13].

Here, we present results of the most comprehensive autoantibody profiling effort undertaken to date in people with established psychotic disorders, using a large and well-characterized sample cohort. We hypothesized that we would identify novel autoantibodies using broad untargeted screening, and that associations between autoantibody expression and clinical features such as psychiatric symptoms, diagnosis, illness course or blood biomarkers of inflammation could be identified. As autoantibody data is known to be highly individual [12], we tested these hypotheses using a stepwise selection procedure. We based this procedure on clinically relevant assumptions, in order to maximize our chances of identifying associations with potential for translation to clinical practice.

## Materials and methods

### Experimental design

We first performed untargeted and broad screening in 24 individuals on group level in eight clinically defined sample pools using planar protein microarrays. Based on these results, our previously unpublished data and literature findings, we designed an antigen panel for the bead-based microarray technology. This panel was used to analyze auto-IgG reactivity in all 461 serum samples.

### Cohort

**Fig. 1:**
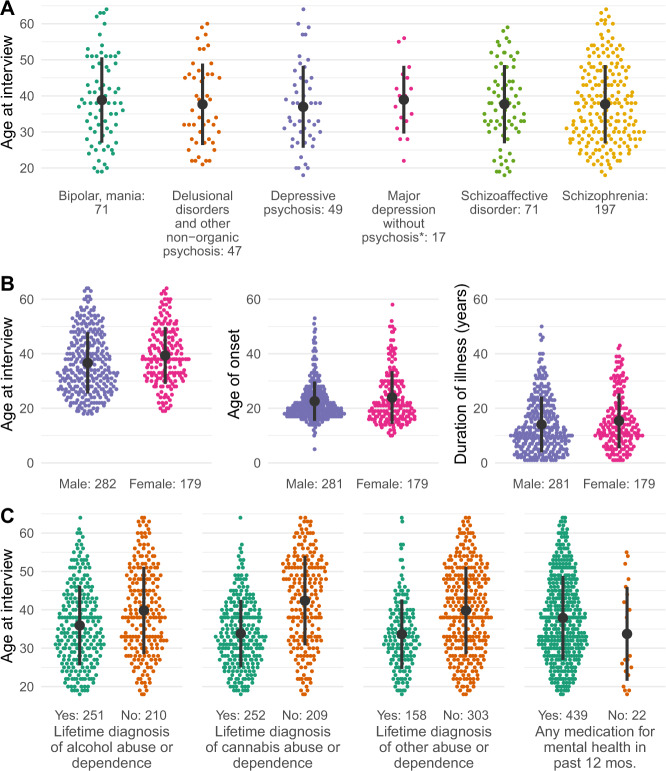
Summary visualization of cohort demographics. **A** Age distribution within DSM-IV diagnoses. **B** Distribution of age, age at illness onset, and duration within males and females. **C** Age distribution by medication use and alcohol, drug, and other abuse. Points and ranges indicate mean and SD. Asterisk (*) symbol indicates these 17 subjects screened positive for psychosis initially but were reclassified to with major depression without psychosis following structured diagnostic interview.

Plasma samples were donated by 461 participants of the 2010 second Australian National Survey of High Impact Psychosis (SHIP), which has been described in detail previously [14]. 204 of these participants were aged 18–34 years and 257 were 35–64 years; 282 (61%) were men. Their mean age was 37.71 (SD = 11.02) years (Fig. 1, Table S1). All participants had screened positive for a psychotic illness. DSM-IV defined diagnoses were confirmed by structured diagnostic interview (Fig. 1, Table S1); participants underwent an in-depth cross-sectional assessment covering a wide range of demographic and clinical features [14]. The research protocol for the study was approved by the Human Research Ethics Committee at the University of Adelaide (H-2018-204), as well as all relevant institutional ethics committees at each catchment site. All participants gave written informed consent.

### Autoantibody profiling

We performed autoantibody profiling in two steps. First, we performed untargeted autoantibody screening using highly multiplex but low-throughput in-house planar protein microarrays to find antigens of interest on the group level. Second, we used high-throughput bead-based microarray technology for targeted autoantibody profiling of individuals.

### Untargeted autoantibody screening on planar microarrays

Proteome-wide untargeted planar protein microarrays consisting of 42100 antigens were used to profile 24 individuals on group level in eight clinically defined plasma pools, as described previously [13] (Supplementary Methods). The antigens included were protein fragments representing regions of gene products from 18,914 Ensembl Gene IDs, based on Ensembl release 92.38, with lowest possible sequence identity to any other gene in the human genome [15]. The detection antibodies used were Alexa 555-conjugated goat anti-chicken IgY (A21437, Invitrogen, Waltham, MA, USA), and Alexa 647-conjugated goat anti-human IgG (A21445, Life Technologies, Carlsbad, CA, USA). The pooling approach was used to enable greater screening width despite low-throughput. Plasma from 24 individuals representing diagnostic and clinical groups within SHIP was selected to form these screening pools, and each pool contained plasma from four individuals (Table S2). The groups represented are: SCZ; BD; severe depression without psychosis; SCZ, chronic course; SCZ, episodic course; SCZ, early onset; SCZ, late onset; SCZ, high negative symptom load. Samples were matched for sex, age, and duration of illness. Where possible, individuals with no history of alcohol- or substance use disorder were selected. Still, due to age and gender matching, the groups severe depression without psychosis; SCZ, chronic course; and SCZ, episodic course, had 50%, 25%, and 25% prevalence of history of alcohol- or substance use disorder, respectively.

### Targeted autoantibody profiling using bead-based microarray technology

Antigens detected in the untargeted screening and antigens indicated by unpublished data and literature analysis (Table S3) were covalently coupled to carboxylated color-coded magnetic microspheres (MagPlex Microspheres, Luminex, Austin, TX, USA), as described elsewhere [16]. Autoantibody profiling using this bead-based assay was performed as previously described [16] (Supplementary Methods). The detection antibody used was R-PE-conjugated anti-human IgG (H10104, Invitrogen, Waltham, MA, USA).

### Data analysis

All data analysis was performed in RStudio [17] using packages base, stats, exact2x2, tidyverse, rlang, ggsignif, ggrepel, pheatmap, UpSetR, cowplot, egg, and rmarkdown.

### Planar arrays

Data from the planar arrays was analyzed using an in-house standard workflow. Raw data from each planar array was obtained as described previously [12]. The raw data was transformed to times standard deviation (SD) using the formula *Times SD* = (*X* − *mean*(*X*))/*SD*(*X*), where times SD is the transformed data, X is the vector of raw data, mean(X) is the mean of the raw data, and SD(X) is the SD of the raw data. Antigens at or exceeding 8 times SD were selected for inclusion in the panel for further analysis.

### Bead-based assay

The raw data from the bead-based experiment was normalized per sample to reduce variance introduced by sample-specific background levels. Normalization was performed using the formula *xMAD* = (*MFI* − *median*(*MFI*))/*mad*(*MFI*), where xMAD is the normalized data, MFI is the raw data of a sample and mad is the median absolute deviation.

A detection cutoff was selected for each antigen using a binning and scoring procedure. The normalized data was partitioned into 16 bins and assigned the score 0-1.5: xMAD < 0, score = 0; 0 ≤ xMAD < 5, score = 0.1; 5 ≤ xMAD < 10, score = 0.2; 10 ≤ xMAD < 15, score = 0.3; …; 65 ≤ xMAD < 70, score = 1.4; 70 ≤ xMAD, score = 1.5.

To set the detection cutoff, an algorithm was used. The following procedure was followed for each antigen: First, the algorithm calculated the kernel density curve of the scored data. Counting from the tallest peak of the density curve, the algorithm found the first point where the slope of the curve passed −0.5. If the x position of the peak was 0.75 or less, the algorithm counted up from the peak. If the x position of the peak was more than 0.75, the algorithm counted down from the peak. The found score was rounded up to the nearest score step, and chosen as the detection cutoff for the antigen. All individuals with scored data at or above the detection cutoff were designated seropositive, and all below the detection cutoff were designated seronegative to the autoantibody in question.

Presence of any individuals with similar autoantibody profiles was examined using complete linkage hierarchical clustering on the Euclidean distance of the autoantibody profiles (R functions dist and hclust in package stats). The results were visualized in a heatmap (R package pheatmap).

Clinical characteristics of high or low reactive individuals were analyzed using Fisher’s exact test on a targeted set of clinical variables (Table S4). Cytokine data was median dichotomized (median included in the lower interval). Cytokine profiles of high or low reactive individuals were analyzed using Fisher’s exact test.

Associations of individual autoantibodies with current or past presence of specific psychiatric symptoms (Table S5) were assessed using three criteria and Fisher’s exact test. First, individual autoantibodies had to be present in at least ten individuals (about 2% of the cohort), to ensure the group size is sufficiently large for potential clinical applicability and to reduce the risk of sporadic associations (*group size criterion*). Second, the prevalence of a specific symptom in the autoantibody-positive group must be at least 85%, to ensure a large effect size of the association (*symptom prevalence criterion*). Third, the prevalence of the symptom must be at least 25% higher in the positive group than in the negative group, to ensure that the association is specific to the positive group (*prevalence ratio criterion*). Lastly, Fisher’s exact test must indicate a statistically significant association after FDR correction (Benjamini–Hochberg).

In addition, a targeted analysis of the clinical characteristics of individuals positive for symptom-associated autoantibodies was conducted using Fisher’s exact test on a selected set of clinical variables (Table S6). Furthermore, cytokine profiles were analyzed using Fisher’s exact test. As these analyses were explorative, no FDR correction for multiple testing was applied.

### Sensitivity analysis

Sensitivity analysis was conducted to assess the effects of cutoff selection on the outcome. Cutoffs were individually varied, with outcome being assessed at each cutoff level.

### Statistical analysis

Hierarchical clustering was performed using the complete linkage method on the Euclidean distance (R package stats [17]). The Shapiro–Wilk test (R package stats [17]) was used for assessment of normality of distributions. Fisher’s exact test was used for analysis of factor variables. R package exact2x2 [18] was used for 2 × 2 tables, while base R [17] was used for higher dimensional tables. All tests were two-tailed (where applicable), and the significance level 0.05 was used. The false discovery rate was controlled in the selection of autoantibodies using the Benjamini–Hochberg procedure (R package stats [17]). False discovery rate due to multiple testing was controlled for in all analyses but the exploratory association with overall autoantibody count and the exploratory further characterization of the selected autoantibodies.

### Molecular visualization

Molecular visualization was carried out using PyMol 2.4.0b0 [19] on model RCSB PDB 5TI9 [20].

## Results

### Broad screening detected autoantibodies to 181 targets in eight clinically defined groups of individuals

The characteristics of the eight clinically defined groups are presented in Table S2. Combining all results, 181 antigens had signals exceeding 8 SD above the mean in at least one group and were therefore classified as seropositive. The resulting autoantibody profiles were highly group-specific, with 89% of the autoantibodies uniquely detected in specific groups (Fig. S1). Of the 181 reactive antigens, 180 were selected (one excluded due to availability), along with 200 additional antigens indicated in previous published and unpublished research, to form the panel for further analysis in individual samples (Table S3).

### Associations of autoantibodies with clinical features

Plasma from 461 individuals (Fig. 1, Table S1) was analysed for IgG reactivity to 381 protein fragments using bead-based technology. The autoantibody landscape of these antigens in the cohort was sparse (sparsity 91.8% among detected autoantibodies), and individual autoantibody profiles were highly unique. Therefore, no prominent clusters of individuals or co-expression patterns could be discerned (Fig. S2), leading us to apply a dichotomous analytical approach.

**Fig. 2:**
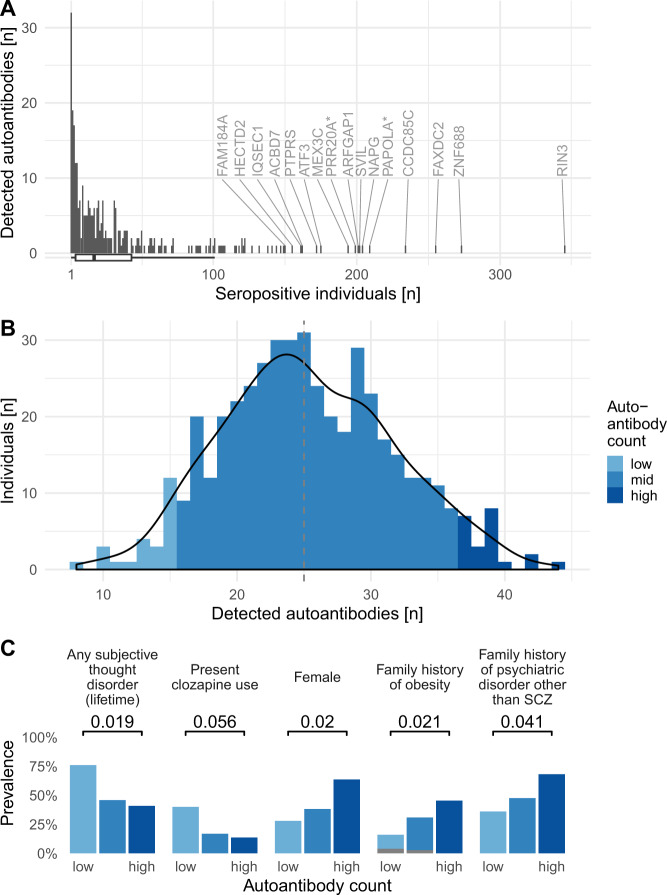
Autoantibody profile of the cohort with associated clinical features. **A** Most detected autoantibodies were found in a small number of individuals only (range 1–345, median = 17, mean = 37 seropositive individuals per autoantibody). No antibody binding was detected for 68 antigens (not shown in figure). Gene names of antigens are indicated for the antigens targeted by the 5% most prevalent autoantibodies. **B** The autoantibody count, i.e., the total number of detected autoantibodies in each sample, deviates slightly from a normal distribution centered on 25 autoantibodies per individual (Shapiro–Wilk test, *W* = 0.993, *p* = 0.035, Fig. S3). We defined individuals with autoantibodies toward fewer than 16 or more than 36 autoantigens (corresponding to the 5th and 95th percentiles; *n* = 25, 22, respectively) as having a low or high autoantibody count, respectively. **C** People with high autoantibody count had higher odds of being female, having a family history of obesity, and having a family history of psychiatric disorder other than Schizophrenia. People with low autoantibody count had higher odds of any present or past subjective thought disorder, and a trend for higher odds of clozapine use. RIN3 Ras and Rab interactor 3, ZNF688 Zinc finger protein 688, FAXDC2 Fatty acid hydroxylase domain-containing protein 2, CCDC85C coiled-coil domain-containing protein 85C, PAPOLA* Poly(A) polymerase alpha, Poly(A) polymerase beta, NAPG, gamma-soluble NSF attachment protein, and SVIL supervillin, ARFGAP1 ADP-ribosylation factor GTPase-activating protein 1, PRR20A* Proline-rich protein 20A, 20B, 20C, 20D, 20E, MEX3C RNA-binding E3 ubiquitin–protein ligase MEX3C, ATF-3 cyclic AMP-dependent transcription factor ATF-3, PTPRS receptor-type tyrosine-protein phosphatase S, ACBD7 Acyl-CoA-binding domain-containing protein 7, IQSEC1 IQ motif and SEC7 domain-containing protein 1, HECTD2 probable E3 ubiquitin–protein ligase HECTD2, FAM184A protein FAM184A, SCZ schizophrenia. Missing data shown in gray.

Autoantibody binding was detected in at least one sample for 312 of the 380 antigens (82%). The distribution of the number of seropositive individuals among detected autoantibodies was left-skewed (range 1–345, median = 17, mean = 37, Fig. 2A) with 50% of detected autoantibodies found in 17 individuals or less. The 16 (5%) most prevalent autoantibody targets included RIN3, ZNF688, and FAXDC2 (Fig. 2A, Table S3). None of these 16 autoantibodies were associated with psychopathology symptoms.

Conversely, the autoantibody count of individuals, i.e., the number of detected autoantibodies in each sample, only deviated slightly from a normal distribution (Shapiro–Wilk test, *W* = 0.99, *p* = 0.035, median 25, Q–Q plot, Fig. S3). We used the 5th and 95th percentiles (seropositive to fewer than 16 or more than 46 autoantibodies, respectively) to define individuals with “low” (*n* = 25) or “high” (*n* = 22) autoantibody count, respectively (Fig. 2B).

### Five clinical features were associated with overall autoantibody count

Further analyses of features of individuals with “high” or “low” autoantibody count revealed associations with five clinical features (Fig. 2C). People with the highest autoantibody count had higher odds of being female (*p* = 0.020, OR = 4.3 (1.3–17)), having a family history of obesity (*p* = 0.021, OR = 5.6 (1.3–28)), and a family history of psychiatric disorders other than SCZ (*p* = 0.041, OR = 3.7 (1.1–14)). Conversely, people with the lowest autoantibody count had higher odds of present or past subjective thought disorder (*p* = 0.019, OR = 4.4 (1.2–18)). Interestingly, we found a trend for increased prevalence of Clozapine treated individuals in the low antibody count group (*p* = 0.056, OR = 4.1 (0.92–20)). Clinical characteristics examined in the targeted analysis are presented in Table S4.

This analysis was evaluated for sensitivity in relation to the applied cutoff levels for high vs. low autoantibody count. Results were insensitive to both increased and decreased stringency of cutoff levels, except for sex and lifetime subjective thought disorder, where loss of significance was observed for decreased stringency. Current Clozapine use was sensitive to cutoff selection in both directions (Fig. S4).

### Six autoantibodies were associated with specific psychopathology symptoms

**Fig. 3:**
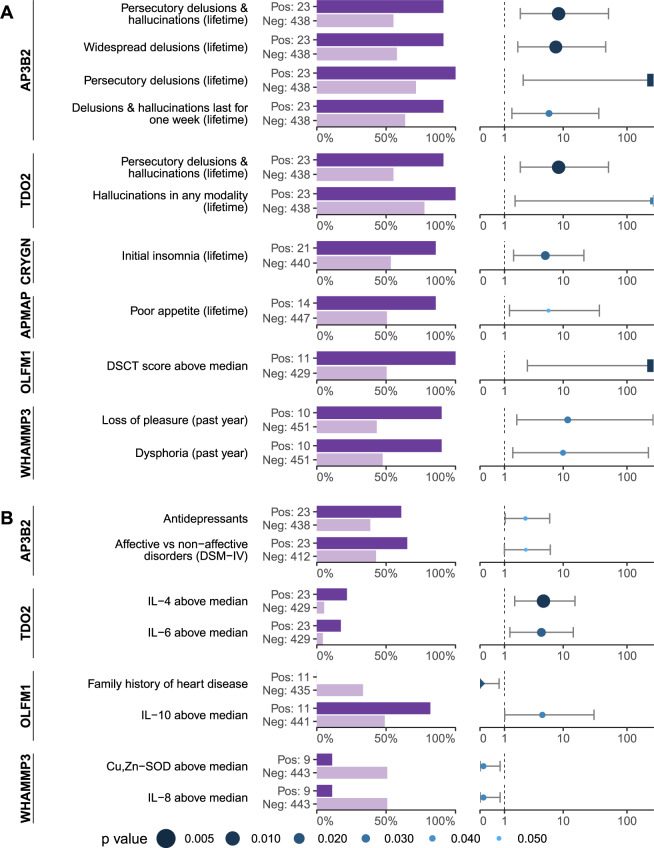
Symptom-, clinical-, and cytokine profiles of individuals with symptom-specific autoantibodies. **A** We found six autoantibodies with high specificity for past or current psychiatric symptoms and meeting selection criteria for group size (*n* ≥ 10), symptom prevalence (symptom present in >85% of seropositive individuals) and symptom prevalence ratio (>25% higher symptom prevalence in seropositive individuals). **B** Some of these symptom-specific autoantibodies were associated with additional clinical and biological features. Bars indicate prevalence of the feature in the seropositive and seronegative groups for the present autoantibody. Dots and confidence intervals indicate odds ratio of the seropositive group. Percentages are within the seropositive/seronegative groups. Half diamonds indicate zero. Half squares indicate infinity. DSCT score median: 39. IL-4 and IL-6 medians: <1 fg/mL. IL-8 median: 3.6 pg/mL. Cu, Zn-SOD median: 333 ng/mL. AP3B2 AP-3 complex subunit beta-2, TDO2 Tryptophan 2,3-dioxygenase, CRYGN Gamma-crystallin N, APMAP Adipocyte plasma membrane-associated protein, OLFM1 Noelin, WHAMMP3 Putative WASP homolog-associated protein with actin, membranes, and microtubules-like protein 1, Pos seropositive individuals, Neg seronegative individuals, IL-4 Interleukin 4, IL-6 Interleukin 6, IL-8 Interleukin 8, IL-10 Interleukin 10, Cu, Zn-SOD, Cu, Zn superoxide dismutase.

We characterized the psychopathology of autoantibody-positive individuals using 205 psychopathology symptoms (Table S5). After application of three selection criteria (minimum of ten seropositive individuals, 85% symptom prevalence in seropositive individuals, and 25% higher symptom prevalence in seropositive individuals, section Data analysis) we found six autoantibodies associated with specific psychopathology symptoms (Fig. 3A, Table 1): anti-AP3B2, anti-TDO2, anti-CRYGN, anti-APMAP, anti-OLFM1, and anti-WHAMMP3 IgG. In particular, anti-AP3B2 was associated with lifetime persecutory delusions (OR = Inf (2.3–Inf)) and lifetime widespread delusions (OR = 7.7 (1.8–46)), anti-TDO2 with lifetime hallucinations in any modality (OR = Inf (1.6–Inf)), and anti-OLFM1 IgG with a digit-symbol coding test score above the median (39) (OR = Inf (2.7–Inf)). The sensitivity of all associations to the selection criteria was also examined using sensitivity analysis. Overall, the symptom associations with anti-AP3B2 and anti-TDO2 IgG were the least sensitive (Figs. S5–7).

**Table 1:** Autoantibodies associated with specific current or past psychopathology symptoms.

Antigen	n (Pos)	Symptom	P(Sx | Pos)	P(Sx | Neg)	PR	*p* value	*q* value	OR (95% CI)
AP3B2	23	Persecutory delusions and hallucinations (lifetime)	91	55	1.7	0.00038	0.01	8.5 (2.0–51)
		Widespread delusions (lifetime)	91	58	1.6	0.00086	0.011	7.7 (1.8–46)
		Persecutory delusions (lifetime)	100	71	1.4	0.0011	0.011	Inf (2.3–Inf)
		Delusions and hallucinations last for 1 week (lifetime)	91	64	1.4	0.006	0.033	6 (1.4–36)
		Affective vs. non-affective disorders (DSM-IV)				0.05		2.5 (1–6.3)
		Antidepressants				0.047		2.5 (1–6.1)
TDO2	23	Persecutory delusions and hallucinations (lifetime)	91	55	1.7	0.00038	0.01	8.5 (2.0–51)
		Hallucinations in any modality (lifetime)	100	78	1.3	0.0068	0.033	Inf (1.6–Inf)
		IL-4 above median				0.01		4.9 (1.6–15)
		IL-6 above median				0.024		4.5 (1.3–14)
CRYGN	21	Initial insomnia (lifetime)	86	53	1.6	0.0032	0.024	5.2 (1.5–21)
APMAP	14	Poor appetite (lifetime)	86	51	1.7	0.012	0.049	5.8 (1.3–37)
OLFM1	11	DSCT score above median	100	50	2.0	0.00091	0.011	Inf (2.7–Inf)
		Family history of heart disease				0.019		0 (0–0.74)
		IL-10 above median				0.036		4.7 (1–30)
WHAMMP3	10	Loss of pleasure (past year)	90	43	2.1	0.0063	0.033	12 (1.7–260)
		Dysphoria (past year)	90	47	1.9	0.0089	0.038	9.9 (1.5–220)
		Cu, Zn-SOD above median				0.037		0.12 (0.0055–0.79)
		IL-8 above median				0.037		0.12 (0.0055–0.79)

Columns n(Pos), P(Sx | Pos), and PR, correspond to the group size, symptom prevalence, and prevalence ratio selection criteria, respectively. P(Sx | Pos), P(Sx | Neg), PR, and *q* value are not applicable to the clinical and biological features associated with psychopathology-specific autoantibodies, and are thus not presented.

*n(Pos)* number of seropositive individuals, *P(Sx* *|* *Pos)* prevalence of symptom in seropositive group, *P(Sx* *|* *Neg)* prevalence of symptom in seronegative group, *PR* prevalence ratio, *OR* odds ratio.

To further characterize the individuals who were seropositive for symptom-specific autoantibodies, we performed a targeted analysis of other clinical characteristics (Table S6), as well as analysis of inflammatory markers (Fig. 3B, Table 1). Notably, anti-AP3B2 positive individuals had increased odds of having an affective psychotic disorder as opposed to a non-affective psychosis (OR = 2.51 (1.0–6.3)), anti-TDO2 positive had increased odds of above-median IL-4 levels (OR = 4.9 (1.6–15)) and IL-6 levels (OR = 4.5 (1.3–14)), and anti-OLFM1 positive had increased odds of IL-10 levels above the cohort median (OR = 4.7 (1.0–30)) and decreased odds of having a family history of heart disease (OR = 0 (0–0.74)).

### Molecular visualization of the IgG–TDO2 binding site

**Fig. 4:**
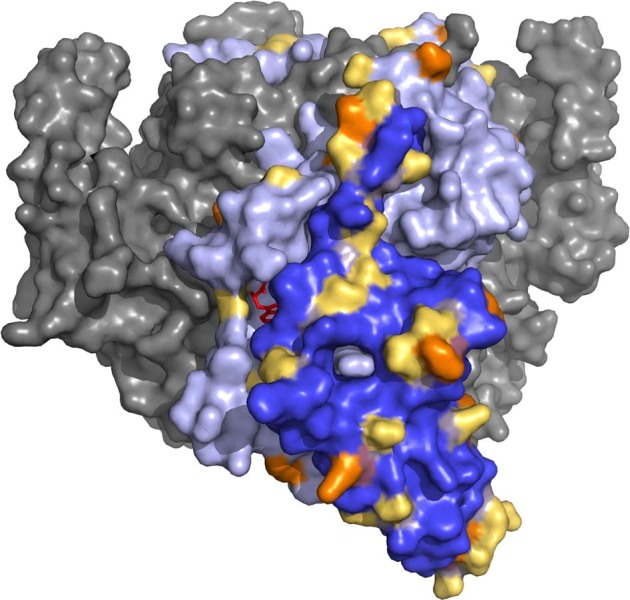
Location of TDO2 antigen on human full-length TDO2 tetramer. The TDO2 antigen covers residue 190–278 (dark blue) of hTDO Chain A. This region covers the L-tryptophan (red) exo binding site as well as 6 of 15 Ub-sites (orange). These 6 sites include all 5 glutamic acid-proximal Ub-sites located on the helix-loop-helix protrusion of Chain A, which is rich in glutamic acid residues (yellow) with negative charge. Both the exo site and the glutamic acid-proximal Ub-sites are of importance in regulating the in vivo half-life of hTDO. Dark blue: Chain A residues corresponding to the TDO2 antigen. Orange: Ub-sites. Yellow: Glutamic acid residues. Light blue: Chain A, not covered by antigen. Ligand, red: L-Tryptophan (in exo site). Gray: Chains B–D. Source: RCSB PDB 5TI9 [20]. TDO2 Tryptophan 2,3-dioxygenase, hTDO human full-length tryptophan 2,3-dioxygenase tetramer, Ub-site ubiquitination site.

To investigate putative autoantibody binding sites, we searched for structural models of the target proteins of the psychopathology-associated autoantibodies. However, only TDO2 had a structural model covering the protein region corresponding to the assay protein fragment. The protein fragment used for anti-TDO2 IgG analysis represented residues 190–278 of TOD2 Chain A and was mapped to a structural model of the protein (Fig. 4).

## Discussion

To our knowledge, this study represents the largest and most comprehensive analysis of peripheral blood autoantibody profiles in people with psychotic disorders to date. We characterized patients with overall high and low autoantibody counts. Additionally, we followed a stepped analysis approach that first identified associations of relatively common autoantibodies with trans-diagnostic psychopathological symptoms before characterizing affected patient groups further using clinical and biological parameters. While autoantibody profiles were highly individual, akin to an “autoimmune fingerprint”, we found that expression of certain autoantibodies are associated with specific clinical features of mental illness, which could have implications for future research and psychiatric practice.

We found some antigens with high general reactivity in the study population, but without apparent associations with features of ill mental health. Several of these reactivities, particularly RIN3 and ZNF688, have been highly prevalent in previous studies of various health and disease contexts [12, 21, 22]; their clinical significance remains unknown and warrants further investigation. People with IgG autoantibodies towards a high number of the antigens in our panel were more likely to be female and to have family histories of obesity and of psychiatric illnesses other than SCZ. Previous studies have shown that females are at increased risk for autoimmune disorders [23] and experience more pronounced self-specific cellular responses to immune challenges [24]. Obesity is a major factor contributing to the onset and progression of autoimmune diseases [25]. Those with autoimmune disorders are more likely to have mental illness [9], with the exception of an inverse association between SCZ and rheumatoid arthritis [26]. Our trend finding of lower autoantibody counts in patients who were treated with clozapine is intriguing. Lower autoantibody counts in this patient group may reflect clozapine’s propensity to induce secondary antibody deficiency [27], but also raises the possibility that suppression of autoimmune processes could be a mechanism of action contributing clozapine’s superior clinical efficacy [28].

We found associations between relatively prevalent autoantibodies to six antigens and specific psychopathology: anti-AP3B2 (persecutory delusions); anti-TDO2 (hallucinations); anti-CRYGN (insomnia); anti-APMAP (poor appetite), anti-OLFM1 (better cognitive performance); and anti-WHAMMP3 IgG (anhedonia and dysphoria).

Anti-AP3B2 autoantibodies were detected in 23 patients who all reported a history of persecutory delusions and who had predominantly affective psychotic disorders. AP3B2 protein is part of the cellular endocytotic machinery, which has previously been implicated in the pathophysiology of psychotic disorders [29]. AP3B2 mRNA levels have been found altered in postmortem brain across a range of psychiatric illnesses and were part of a gene co-expression network associated with synaptic changes across multiple diagnoses [6].

Anti-TDO2 autoantibodies were detected in 23 patients who all had experienced hallucinations. In addition, we found indications that anti-TDO2-positive patients were more likely to display increased levels of the pro-inflammatory cytokines IL-6 and IL-4. The TDO2 protein is a rate limiting enzyme of the kynurenine (KYN) pathway whose neuro-modulatory end-product, kynurenic acid (KA) has been reported as increased in samples from people with psychosis [30]. Activation of the KYN pathway co-occurs with peripheral inflammatory marker increases following immune challenges [31]. A specific symptom association of elevated KA with hallucinations has been reported in a previous study of people with mild dementia [32]. The region covered by the protein fragment is important in protein degradation, as it contains all five glutamic acid-proximal ubiquitination sites of TDO2 [20]. Thereby, autoantibody-antigen interaction could prevent ubiquitination-dependent proteasomal degradation, in turn leading to increased activity of the kynurenine pathway and accumulation of neurotoxic metabolites including kynurenic acid. In conclusion, our molecular mapping suggests that anti-TDO2 IgG-antigen interaction could result in the increased production of KA through inhibition of ubiquitin-dependent proteasomal degradation, a hypothesis that is testable in future studies.

The 11 people with anti-OLFM1 autoantibodies all had above-median cognitive performance on the DCST test. In addition, we found indications that they had above-median levels of the anti-inflammatory cytokine IL-10 and that they were less likely to have family histories of heart disease. OLFM1 protein is highly expressed throughout the brain and enriched in synaptosomes, where it is thought to regulate receptor trafficking to the synaptic membrane microdomain [33]. In postmortem tissue from the dorsolateral prefrontal cortex, OLFM1 mRNA was found increased in people with chronic SCZ [34]. Moreover, OLFM1 is part of the disrupted in schizophrenia 1 (DISC1) protein interactome [35], variations of which were shown to increase the risk of poorer childhood cognitive performance in people who later developed SCZ [36]. Our findings raise the intriguing possibility, to be investigated in future studies, that OLFM1-autoimmunity could be protective against such effects.

Five of the six autoantibodies we found associated with specific psychopathologies were discovered in the untargeted autoantibody screening on planar arrays, and hence represent novel findings. It is notable that neuroreceptor-autoantibodies such as anti-NMDAR, which we had included on our bead array panel given their prominence in the psychiatric literature, were not associated with specific psychopathological features investigated in our stepped analysis. However, it is possible that the presence of neuroreceptor autoantibodies is linked to other important clinical parameters such as course of illness or treatment response [37], which were not included in our initial analysis step.

Our study has some limitations. First, this is a cross-sectional investigation in people with established and often chronic mental illness who mostly had extensive pharmacologic treatment histories and high levels of physical co-morbidity and substance use. While we have included these factors in our analyses, their potential impact on autoantibody-symptom associations cannot fully be controlled for in our study design. Second, repeated measures of autoantibody levels over time are warranted to ascertain whether candidate markers are indeed longitudinally stable in individuals or whether they are subject to fluctuation and environmental influence. Third, discovery studies are exploratory by design and findings have to be interpreted in light of this. Replication and validation studies are needed to corroborate the findings. Fourth, discovery studies are constrained by technical limitations. Here, these include the experimental selection of antigen representations, the relatively small number of individuals whose plasma was subjected to initial broad screening, and the depth and precision of the phenotypic characterization. Fifth, this study was performed using peripheral plasma. Therefore, it is not clear whether detected autoantibody profiles are representative of those in cerebrospinal fluid [8]. However, previous work using our in-house bead-based arrays has showed that ~50% of detected autoantibodies in plasma correlate well (Spearman’s rho = 0.49–0.92) with levels in cerebrospinal fluid [38]. Sixth, we felt that a psychopathological grid would yield the most innovative and fine-grained mapping of autoimmunity to expressions of mental illness. The SHIP dataset provided a sufficiently rich account of psychopathological characterizations to support our approach. However, a different initial antibody selection strategy that included DSM diagnoses, features of functioning, course of illness, or treatment response may have steered our study into a different direction. Inclusion of a wider range of clinical parameters in our initial selection strategy would have exacerbated the issue of multiple comparisons, reducing the power of the study.

In conclusion, our study suggests that autoantibody profiling can reveal potentially important associations of autoantibodies with specific symptoms and clinical features of mental illness. If findings are replicated and further developed, autoantibody assessment could be incorporated into pathways towards personalized clinical psychiatry, aiming for better patient stratification to improve diagnosis and treatments.

## Supplementary information

Supplemental material

Table S3

## Data Availability

Data cannot be shared publicly as it contains sensitive personal information which is protected by the GDPR. Data are available on request from the SciLifeLab Data Repository (10.17044/scilifelab.16451112) for researchers who meet the criteria for access to sensitive personal data.

## Code availability

Code cannot be shared publicly as sensitive personal information which is protected by the GDPR can be inferred. Code is available on request from the SciLifeLab Data Repository (10.17044/scilifelab.16451112) for researchers who meet the criteria for access to sensitive personal data.
