# Supplementary material for: Autoantibody profiles associated with clinical features in psychotic disorders
Source: Transl Psychiatry. 2021 Sep 13;11:474. doi: 10.1038/s41398-021-01596-0 (PMC8438048; doi:10.1038/s41398-021-01596-0)
Supplement: Supplementary file 1 — Supplemental material [file 41398_2021_1596_MOESM1_ESM.docx]

**Autoantibody profiles associated with clinical features in psychotic disorders**

**Supplementary information**

**Authors:**

August Jernbom Falk^1^
Cherrie Galletly^2,3,4^
David Just^1^Catherine Toben^2^
Bernhard T. Baune^2,5,6,7,8,9,10^
Scott R. Clark^2^
Dennis Liu^2,3^Peter Nilsson^1^
Anna Månberg^1^
K. Oliver Schubert^2,3^

**Affiliations:**

^1^Department of Protein Science, KTH Royal Institute of Technology, SciLifeLab, Stockholm, Sweden

^2^Discipline of Psychiatry, Adelaide Medical School, University of Adelaide, Adelaide, SA, Australia

^3^Northern Adelaide Mental Health Services, SA Health, Adelaide, SA, Australia

^4^Ramsay Health Care (SA) Mental Health, The Adelaide Clinic, Adelaide, SA, Australia

^5^Department of Mental Health, University of Münster, Münster, Germany

^6^Lab division of Molecular Neurobiology of Mental Health, University of Münster, Münster, Germany

^7^Department of Psychiatry, Melbourne Medical School, The University of Melbourne, Melbourne, VIC, Australia

^8^The Florey Institute of Neuroscience and Mental Health, The University of Melbourne, Parkville, VIC, Australia

^9^Department of Psychiatry, SUNY Upstate Medical University, Syracuse, NY, USA

^10^School of Psychiatry, University of New South Wales, Sydney, NSW, Australia

**Corresponding authors:**

August Jernbom Falk
SciLifeLab, Department of Protein Science, Division of Affinity Proteomics, KTH Royal Institute of Technology
Tomtebodavägen 23 A
171 65 Solna, Stockholm, Sweden
august.jernbom@scilifelab.se

A/Professor K. Oliver Schubert
Discipline of Psychiatry, Adelaide Medical School, University of Adelaide
Level 6, Adelaide Medical and Health Science Building
North Terrace, Adelaide 5000, South Australia, Australia
Oliver.schubert@adelaide.edu.au

Table of Contents

**Supplementary Methods3**

Untargeted screening on planar microarrays3

Autoantibody profiling using a bead-based microarray3

Cytokine measures3

Multiplex enhanced sensitivity cytometric bead array (esCBA) assay for pro and anti-inflammatory cytokines3

Oxidative stress marker copper zinc superoxide dismutase (Cu/Zn SOD) ELISA3

**Supplementary Figures4**

Figure S1: Autoantibody profile overlap of sample pools analyzed on planar arrays 4

Figure S2: Autoantibody landscape of cohort 5

Figure S3: Normal Q-Q plot of autoantibody count 6

Figure S4: Sensitivity analysis of associations to high and low autoantibody count 7

Figure S5: Sensitivity analysis of associations of symptoms to selected autoantibodies: Group size criterion 8

Figure S6: Sensitivity analysis of associations of symptoms to selected autoantibodies: Symptom prevalence criterion 9

Figure S7: Sensitivity analysis of associations of symptoms to selected autoantibodies: Prevalence ratio criterion 10

**Supplementary Tables11**

Table S1: Cohort demographics.11

Table S2: Demographics of sample pools.14

Table S3: Details of antigens in the panel for analysis of individual samples.15

Table S4: Cohort demographics of variables examined in targeted analysis of individuals with high or low autoantibody count16

Table S5: Cohort demographics of psychopathological symptoms examined for associations with detected autoantibodies19

Table S6: Cohort demographics of variables examined in secondary analysis of individuals with symptom-specific autoantibodies27

**References29**

# **Supplementary Methods**

## **Untargeted screening on planar microarrays**

Untargeted screening was performed as described previously ^1^. The plasma pools were diluted 1:100 in assay buffer (1x phosphate-buffered saline (PBS) with 1% v/v Tween-20, 3% w/v bovine serum albumin, 5% w/v non-fat milk powder, and 160 µg/ml hexahistidyl-albumin binding protein fusion tag (His_6_ABP, in-house)) and incubated for 15 min. After incubation, 100 µl diluted plasma was applied to the microarray slides for 1 h. Slides were washed four times in 1% PBS-T (PBS with 1% v/v Tween-20), and detection of microarray spots was performed with chicken anti-His_6_ABP IgY (in-house, 1:40000 in 0.1% PBS-T). After 1 h incubation and three washes in 1% PBS-T, fluorescently labeled detection antibodies (Alexa 555-conjugated goat anti-chicken IgY (A21437, Invitrogen, Waltham, MA, USA), and Alexa 647-conjugated goat anti-human IgG (A21445, Life Technologies, Carlsbad, CA, USA), both 1:15000 in 0.1% PBS-T), were added and incubated for 1 h. Readout was performed at in a microarray scanner (G2565BA, Agilent, Santa Clara, CA, USA, red channel 950 nm, green channel 700 nm), and images were analyzed using GenePix Pro 5.1 (Molecular Devices, Sunnyvale, CA, USA).

## **Autoantibody profiling using a bead-based microarray**

Autoantibody analysis with the bead-based assay was performed as previously described ^2^. In brief, the bead array was distributed in two 384-well polystyrene microtiter plates, and samples were diluted 1:250 in assay buffer (same as above) in 96-well plates. After 1 h pre-incubation, 45 µl of each diluted sample was added to the bead plates. The plates were then incubated for 2 h, washed in 0.05 % PBS-T, and incubated for 10 min in 0.2 % PFA (PBS with 0.2 % v/v paraformaldehyde). After washing in 0.05 % PBS-T, detection antibody was added (0.4 µg/ml R-PE-conjugated anti-human IgG, H10104, Invitrogen, diluted 1:500 in 0.05% PBS-T), and the plates were incubated for 30 min. Detection was performed using Luminex FLEXMAP 3D instruments (Luminex, Austin, TX, USA) and antibody levels reported as relative levels (AU).

**Cytokine measures**

### **Multiplex enhanced sensitivity cytometric bead array (esCBA) assay for pro and anti-inflammatory cytokines**

Simultaneous measurement of multiple cytokines (IL-1b, IL-2, IL-4, IL-6, IL-8, IL-10, IL-12, IFNƔ and TNFα) was conducted using BD Biosciences human esCBA kits (BD Biosciences, San Jose CA) according to modified manufacturer instructions. This included an 11-point generated standard curve and duplicate samples assayed at neat. Acquisition was performed on a Becton Dickinson LSR Fortessa using FACS Diva Software version 8.0 (BD Biosciences, San Diego, CA, USA) and detectors set with the following parameters: PE; 561nm laser excitation, 585/15 bandpass, APC; 640nm laser excitation, 670/14 bandpass and APC-Cy7; 640nm laser excitation, 780/60 bandpass. Sensitivity and daily performance was checked using BD FACSDiva^TM^ CS&T research beads (Becton Dickinson, Franklin Lakes, NJ, USA)). The BD CBA FCAP Array 3 software (Soft Flow Inc., Pecs, Hungary) calculated each cytokine’s mean fluorescent intensity (MFI) using the PE conjugated detector antibody. The protein concentration was modelled as a function of the MFI and a standard curve for each cytokine was generated. Thereafter a 5-parameter logistic regression model implemented the generated standard curves to calculate the nine cytokine concentrations

### **Oxidative stress marker copper zinc superoxide dismutase (Cu/Zn SOD) ELISA**

Copper zinc superoxide dismutase (Cu/Zn SOD) was analysed according to manufacturer instructions for the human Abcam ELISA kit (Abcam, Cambridge, UK). Briefly duplicate plasma samples were measured at absorbance wavelength of 450nm with correction at 570nm on the Biotek Synergy MX plate reader. Graphpad Prism6 v7 analysed generated data calculating unknown average values using nonlinear regression models with corrections for background readings. Manufacturer’s reported inter and intra assay accuracy was 5.8% and 5.1% respectively.

# **Supplementary Figures**


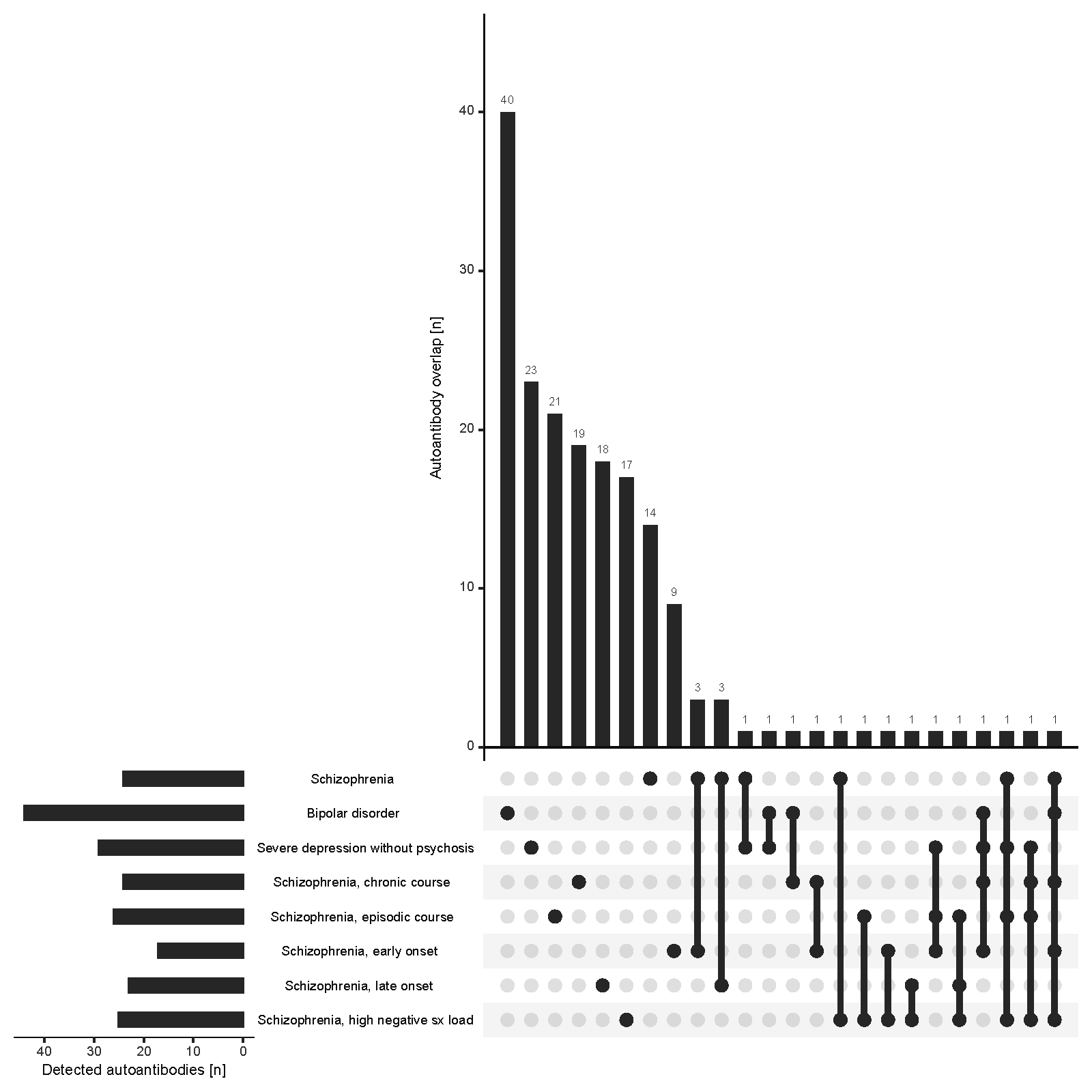


**Figure S1: Autoantibody profile overlap of sample pools analyzed on planar arrays.** Autoantibody profiles were highly unique; of the 181 detected autoantibodies, 89% were unique to one pool only.**Figure S2: Autoantibody landscape of cohort.** The autoantibody landscape of the cohort was highly (91.8%) sparse among detected autoantibodies. As indicated by the dendrogram, individual autoantibody profiles were highly unique. No prominent clusters of individuals could be discerned using Hierarchical clustering with Euclidean distance on the complete set of detected autoantibodies. Only autoantibodies detected in at least one individual were included in the clustering and display. **Figure S3: Normal Q-Q plot of autoantibody count**. Although the autoantibody count deviates from a normal distribution (Shapiro-Wilk test, W = 0.993, p = 0.035), the effect size of this deviation was very small. **Figure S4: Sensitivity analysis of associations to high and low autoantibody count.** The numbers in the x-axis legend refers to the number of reactive antigens per sample with one reactive antigen added in each group left to right. Associations of high and low autoantibody count to family history of obesity and to family history of psychiatric disorders other than Schizophrenia were insensitive to both increasing and decreasing cutoff stringency. The associations with sex and lifetime subjective thought disorder were insensitive to increasing stringency only. The association to Clozapine use was sensitive to cut-off selection.


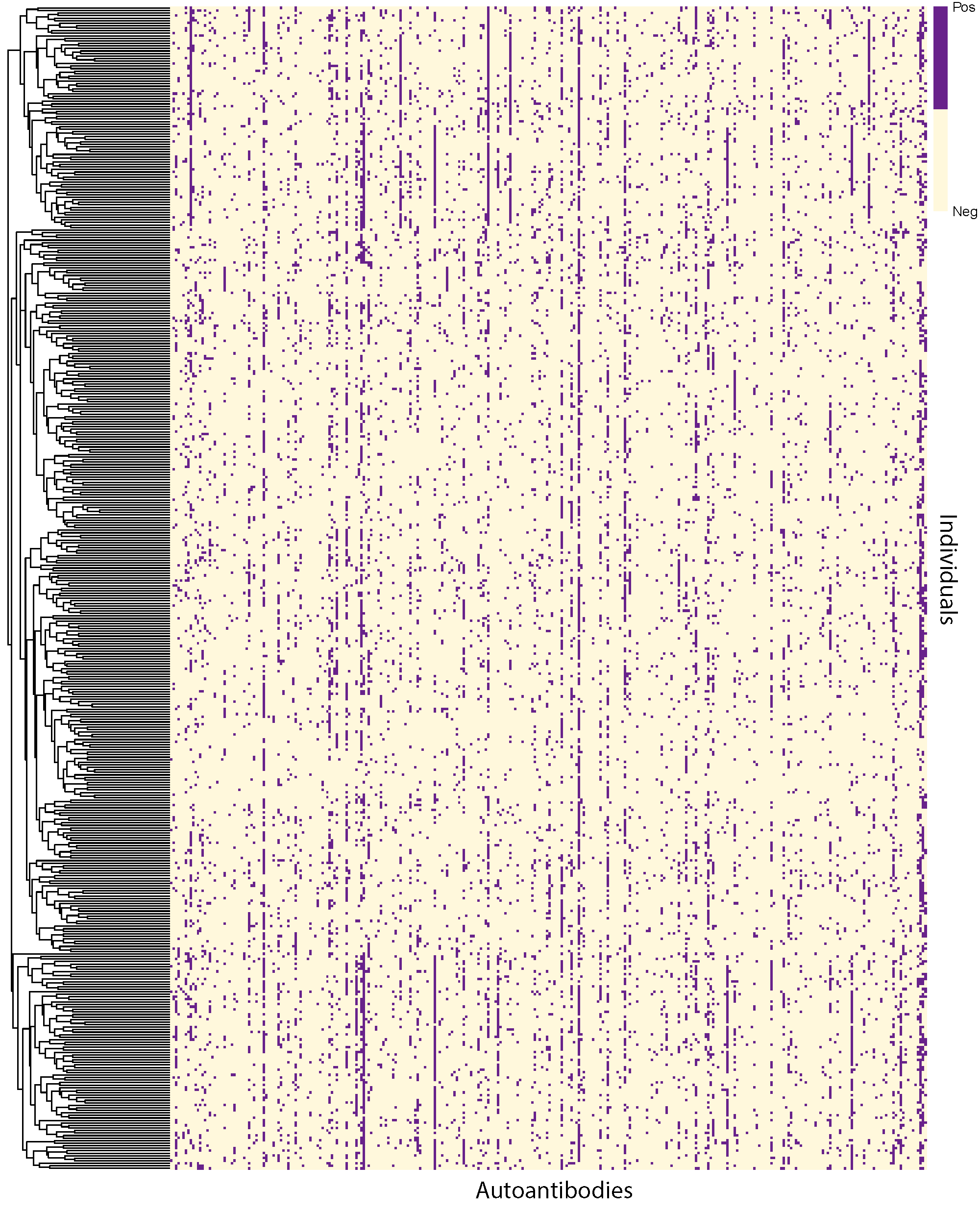

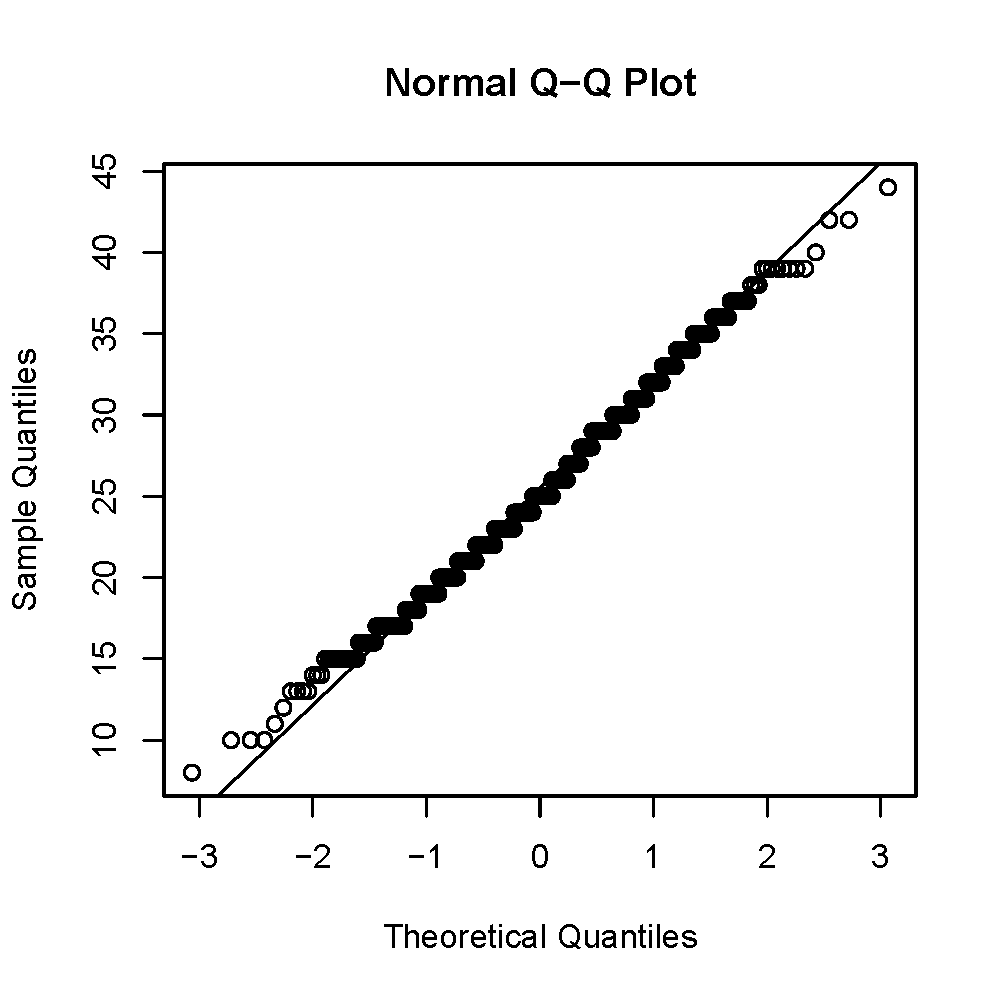

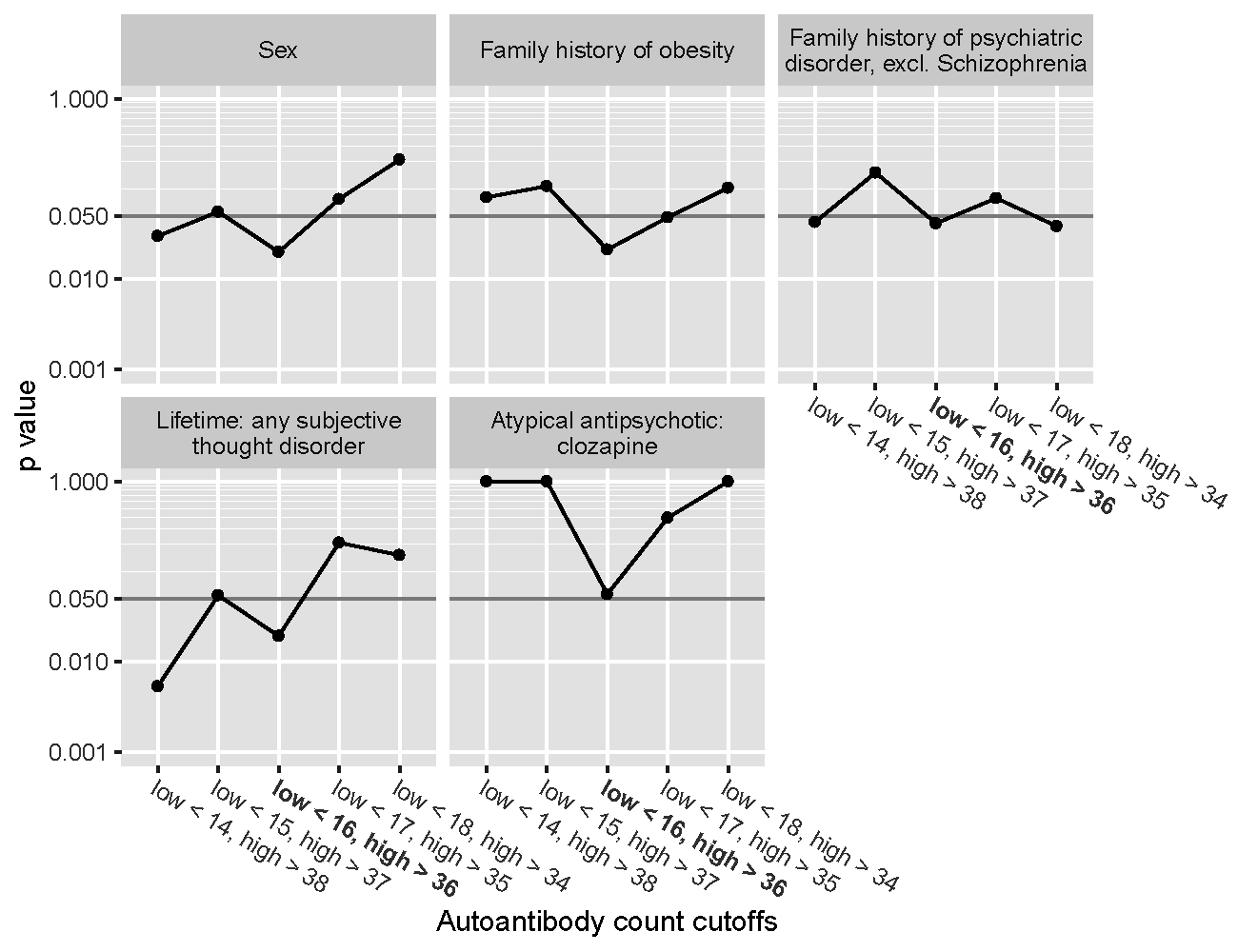

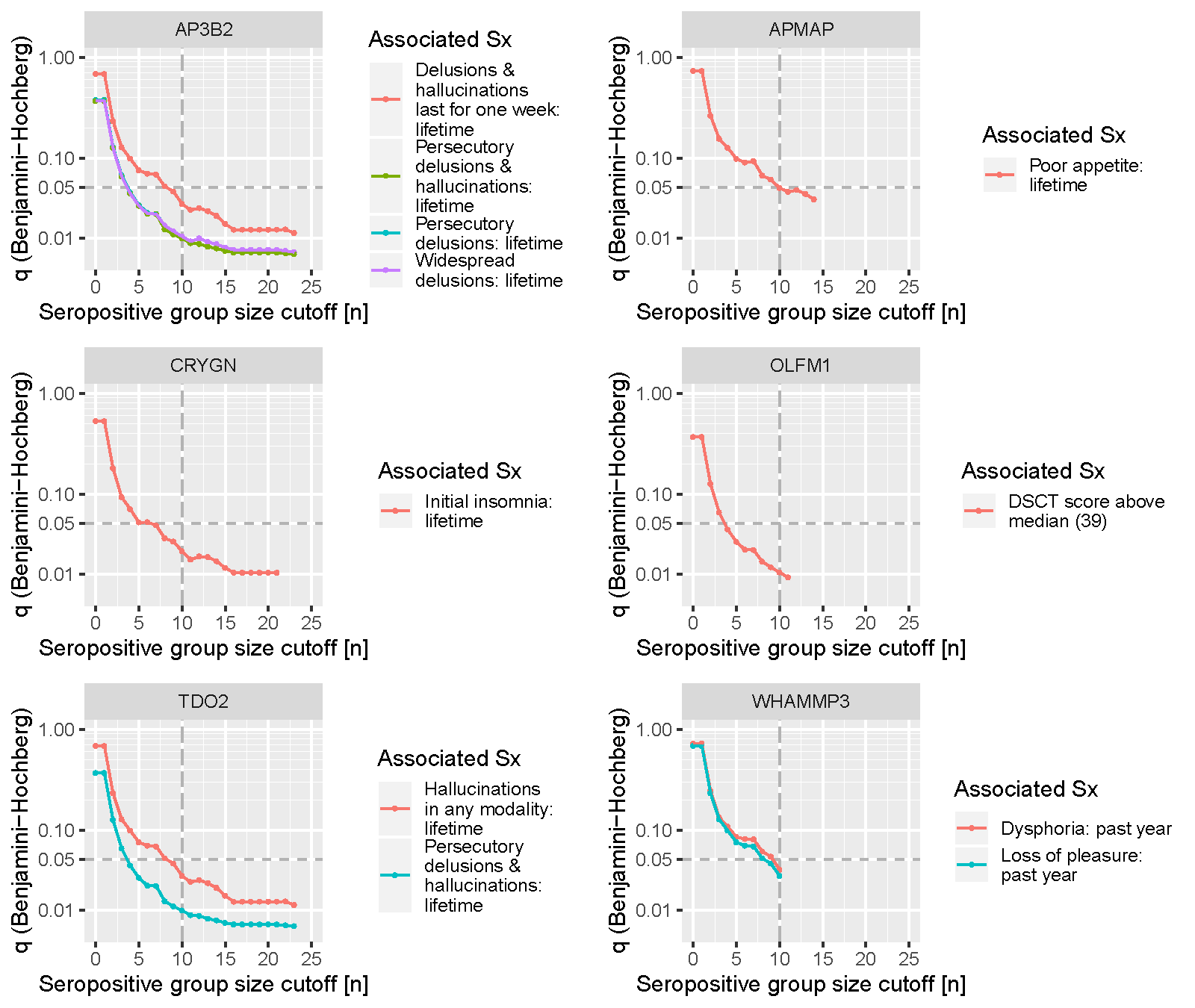


**Figure S5: Sensitivity analysis of associations of symptoms to selected autoantibodies: Group size criterion.** The x axis shows the cutoff for number of seropositive individuals for the autoantibody. Line segments are shown where the combination of autoantibody and symptom were selected. The symptom prevalence and prevalence ratio criteria were kept constant at 0.85 and 1.25, respectively.


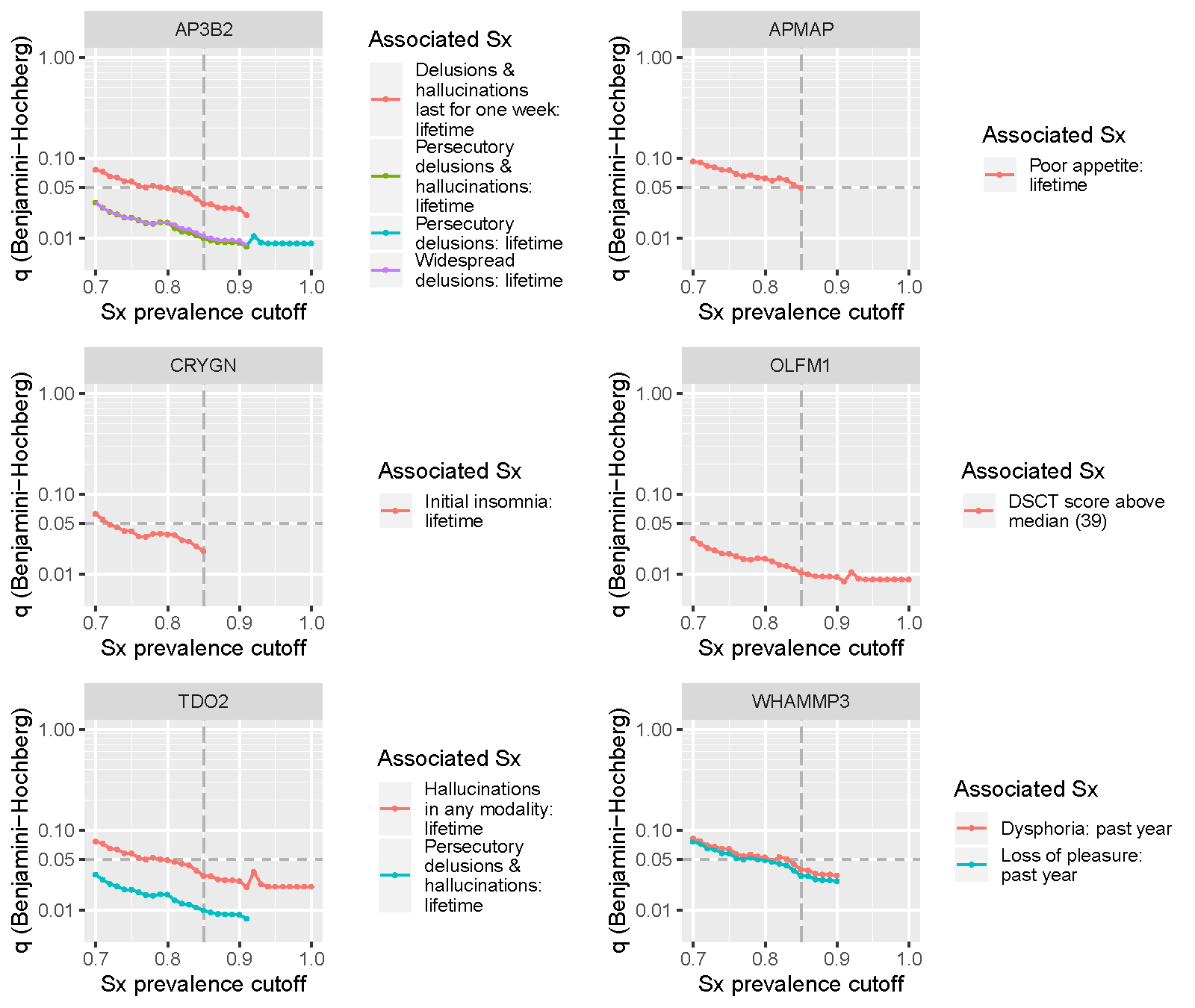


**Figure S6: Sensitivity analysis of associations of symptoms to selected autoantibodies: Symptom prevalence criterion.** The x axis shows the cutoff for symptom prevalence in the seropositive group. Line segments are shown where the combination of autoantibody and symptom were selected. The group size and prevalence ratio criteria were kept constant at 10 and 1.25, respectively.


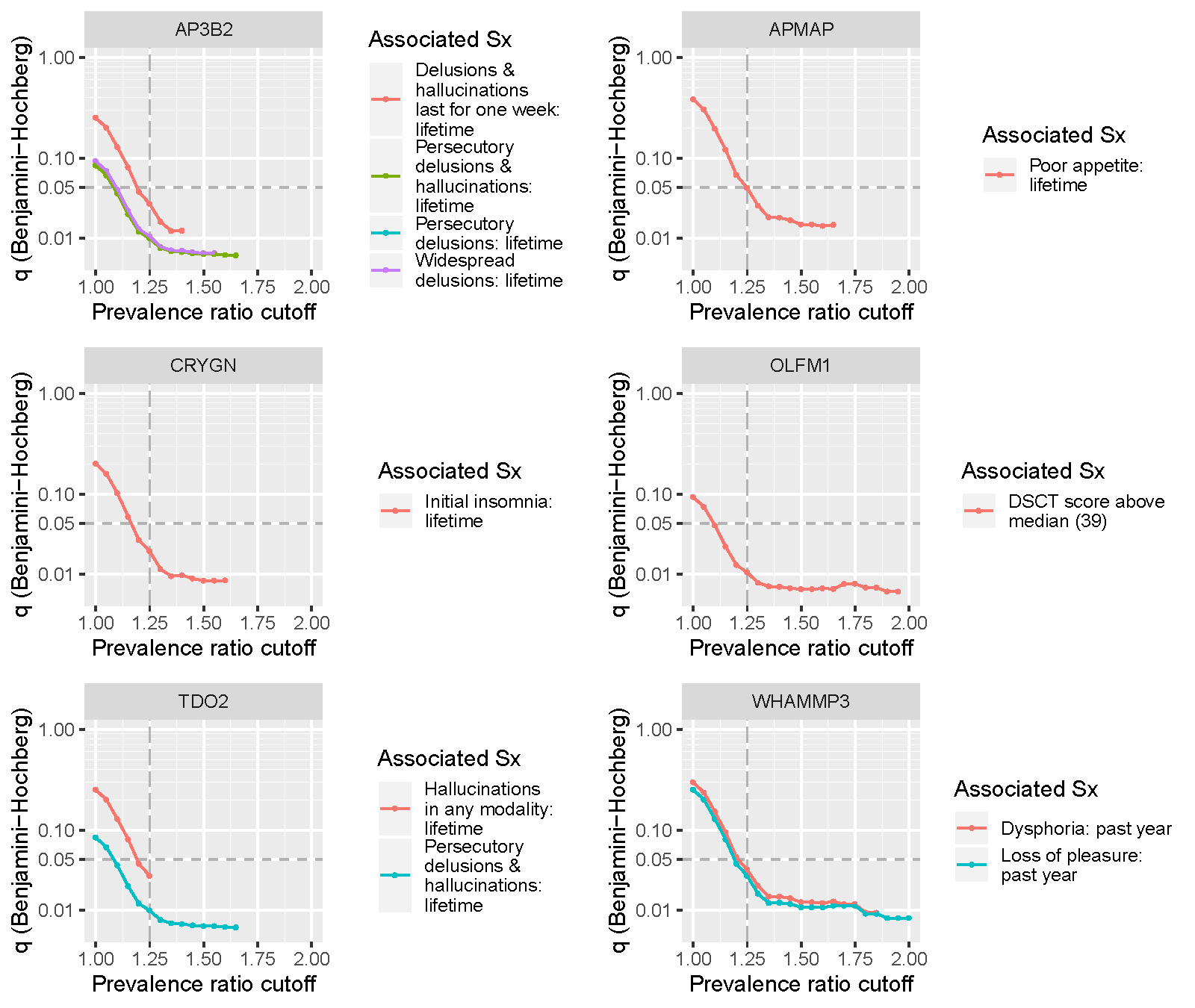


**Figure S7: Sensitivity analysis of associations of symptoms to selected autoantibodies: Prevalence ratio criterion.** The x axis shows the cutoff for symptom prevalence ratio (prevalence in seropositive group/prevalence in seronegative group). Line segments are shown where the combination of autoantibody and symptom were selected. The group size and symptom prevalence criteria were kept constant at 10 and 0.85, respectively.

# **Supplementary Tables**

**Table S1: Cohort demographics.**

| **Variable** | **Mean (SD)** | **No data, n** |
| --- | --- | --- |
| Age at interview | 37.7 (11) | 0 |
| Age of onset | 23.1 (8.2) | 1 |
| Duration of illness (years) | 14.6 (10.2) | 1 |
| **Variable** | **Category** | **n (%)** |
| Sex | Male | 282 (61) |
|  | Female | 179 (39) |
| Diagnosis (DSM-IV) | Screen-positive for psychosis but did not meet full DSM-IV criteria | 9 (2) |
|  | Schizophrenia | 197 (43) |
|  | Schizoaffective disorder | 71 (15) |
|  | Major depression without psychosis | 17 (4) |
|  | Depressive psychosis | 49 (11) |
|  | Delusional disorders and other non-organic psychosis | 47 (10) |
|  | Bipolar, mania | 71 (15) |
| Any medications for mental health in past 12 months | Yes | 439 (95) |
|  | No | 22 (5) |
| Lifetime diagnosis of alcohol abuse or dependence | Yes | 251 (54) |
|  | No | 210 (46) |
| Lifetime diagnosis of cannabis abuse or dependence | Yes | 252 (55) |
|  | No | 209 (45) |
| Lifetime diagnosis of other abuse or dependence | Yes | 158 (34) |
|  | No | 303 (66) |
| Any hallucinations, lifetime | Yes | 368 (80) |
|  | No | 93 (20) |
| Any delusions, lifetime | Yes | 387 (84) |
|  | No | 74 (16) |
| Any subjective thought disorder, lifetime | Yes | 218 (47) |
|  | No | 243 (53) |
| Any depressive symptoms, lifetime | Yes | 369 (80) |
|  | No | 92 (20) |
| Symptoms of mania, lifetime | Yes | 178 (39) |
|  | No | 283 (61) |
| NART Fullscale IQ - median dichotomy | Low | 200 (43) |
|  | High | 215 (47) |
|  | No data | 46 (10) |

| **Variable** | **Category** | **n (%)** |
| --- | --- | --- |
| DSCT - median dichotomy | Low | 213 (46) |
|  | High | 227 (49) |
|  | No data | 21 (5) |
| Family history of obesity | Yes | 129 (28) |
|  | No | 319 (69) |
|  | No data | 13 (3) |
| Family history of psychiatric disorder other than Schizophrenia | No | 240 (52) |
|  | Yes | 221 (48) |
| Present clozapine use | Yes | 83 (18) |
|  | No | 378 (82) |
| Persecutory delusions & hallucinations, lifetime | Yes | 263 (57) |
|  | No | 198 (43) |
| Widespread delusions, lifetime | Yes | 274 (59) |
|  | No | 187 (41) |
| Persecutory delusions, lifetime | Yes | 336 (73) |
|  | No | 125 (27) |
| Delusions and hallucinations last for one week, lifetime | Yes | 300 (65) |
|  | No | 161 (35) |
| Hallucinations in any modality, lifetime | Yes | 363 (79) |
|  | No | 98 (21) |
| Initial insomnia, lifetime | Yes | 253 (55) |
|  | No | 208 (45) |
| Poor appetite, lifetime | Yes | 238 (52) |
|  | No | 223 (48) |
| Loss of pleasure, lifetime | Yes | 328 (71) |
|  | No | 133 (29) |
| Dysphoria, lifetime | Yes | 366 (79) |
|  | No | 95 (21) |
| Present antidepressant use | Yes | 183 (40) |
|  | No | 278 (60) |
| Affective vs non-affective disorders (DSM-IV) | Non-affective | 244 (53) |
|  | Affective | 191 (41) |
|  | No data | 26 (6) |
| IL-4 | Pos | 28 (6) |
|  | Neg | 424 (92) |
|  | No data | 9 (2) |
| IL-6 | Pos | 23 (5) |
|  | Neg | 429 (93) |
|  | No data | 9 (2) |

| **Variable** | **Category** | **n (%)** |
| --- | --- | --- |
| Family history of heart disease | Yes | 145 (31) |
|  | No | 301 (65) |
|  | No data | 15 (3) |
| IL-10 | Pos | 225 (49) |
|  | Neg | 227 (49) |
|  | No data | 9 (2) |
| Cu,Zn-SOD | Pos | 226 (49) |
|  | Neg | 226 (49) |
|  | No data | 9 (2) |
| IL-8 | Pos | 226 (49) |
|  | Neg | 226 (49) |
|  | No data | 9 (2) |

**Table S2: Demographics of sample pools.**

| **Pool** | **Clinical characteristic** | **DSM diagnosis** | **Sex** | **Age** | **Age of onset (yrs)** | **Lifetime Hx of any alcohol or drug use disorder** | **Duration of illness (yrs)** | **Number of negative Sx (Carpenter)** | **Comment** |
| --- | --- | --- | --- | --- | --- | --- | --- | --- | --- |
| 1 | Schizophrenia | SCZ | Male | 30 |  | No |  |  |  |
|  |  | SCZ | Male | 28 |  | No |  |  |  |
|  |  | SCZ | Female | 29 |  | No |  |  |  |
|  |  | SCZ | Female | 32 |  | No |  |  |  |
| 2 | Bipolar disorder | Bipolar | Male | 26 |  | No |  |  |  |
|  |  | Bipolar | Male | 25 |  | No |  |  |  |
|  |  | Bipolar | Female | 30 |  | No |  |  |  |
|  |  | Bipolar | Female | 33 |  | No |  |  |  |
| 3 | Severe depression without psychosis | MDD | Male | 22 |  | Alcohol |  |  |  |
|  |  | MDD | Male | 33 |  | No |  |  |  |
|  |  | MDD | Female | 28 |  | No |  |  |  |
|  |  | MDD | Female | 28 |  | Alcohol and drugs |  |  |  |
| 4 | Schizophrenia, chronic course | SCZ | Male | 34 |  | No | 17 |  | Chronic course (Non deteriorating) |
|  |  | SCZ | Male | 21 |  | No | 4 |  | Chronic course (Non deteriorating) |
|  |  | SCZ | Female | 33 |  | Cannabis | 14 |  | Chronic course (Non deteriorating) |
|  |  | SCZ | Female | 22 |  | No | 8 |  | Chronic course (Non deteriorating) |
| 5 | Schizophrenia, episodic course | SCZ | Male | 33 |  | No | 7 |  | Episodic course (good recovery in between) |
|  |  | SCZ | Male | 32 |  | No | 10 |  | Episodic course (good recovery in between) |
|  |  | SCZ | Female | 21 |  | No | 6 |  | Episodic course (good recovery in between) |
|  |  | SCZ | Female | 40 |  | Cannabis | 22 |  | Episodic course (good recovery in between) |
| 6 | Schizophrenia, early onset | SCZ | Male | 21 | 17 | No | 4 |  | Onset < 21 |
|  |  | SCZ | Male | 28 | 20 | No | 8 |  | Onset < 21 |
|  |  | SCZ | Female | 24 | 14 | No | 10 |  | Onset < 21 |
|  |  | SCZ | Female | 21 | 15 | No | 6 |  | Onset < 21 |
| 7 | Schizophrenia, late onset | SCZ | Male | 49 | 31 | No | 17 |  | Onset >28 |
|  |  | SCZ | Male | 30 | 28 | No | 2 |  | Onset >28 |
|  |  | SCZ | Female | 43 | 33 | No | 10 |  | Onset >28 |
|  |  | SCZ | Female | 43 | 38 | No | 5 |  | Onset >28 |
| 8 | Schizophrenia, high negative Sx load | SCZ | Male | 32 | 19 | No | 13 | 5 | High negative Sx |
|  |  | SCZ | Male | 33 | 26 | No | 7 | 6 | High negative Sx |
|  |  | SCZ | Female | 22 | 14 | No | 8 | 6 | High negative Sx |
|  |  | SCZ | Female | 21 | 14 | No | 6 | 5 | High negative Sx |

**Table S3: Details of reactive antigens in the panel for analysis of individual samples.**

Table provided in separate Excel file.

**Table S4: Cohort demographics of variables examined in targeted analysis of individuals with high or low autoantibody count.** The level NA refers to missing data.

|  |  | **Autoantibody count** | | |
| --- | --- | --- | --- | --- |
| **Variable** | **Level** | **Low (n)** | **Mid (n)** | **High (n)** |
| Affective vs non-affective disorders (DSM-IV) | Affective | 14 | 166 | 11 |
|  | Non-affective | 10 | 225 | 9 |
|  | NA | 1 | 23 | 2 |
| Affective vs non-affective disorders (ICD-10) | Affective | 11 | 148 | 9 |
|  | Non-affective | 11 | 227 | 10 |
|  | NA | 3 | 39 | 3 |
| Age at interview | 18-34 | 13 | 184 | 7 |
|  | 35-64 | 12 | 230 | 15 |
| Antidepressants | No | 15 | 250 | 13 |
|  | Yes | 10 | 164 | 9 |
| Any Cardiovascular disease | No | 24 | 372 | 19 |
|  | Yes | 1 | 42 | 3 |
| Atypical antipsychotic: clozapine | No | 15 | 344 | 19 |
|  | Yes | 10 | 70 | 3 |
| Atypical antipsychotics | No | 6 | 110 | 8 |
|  | Yes | 19 | 304 | 14 |
| BMI -criteria from World Health Organisation | No measurement | NA | 3 | NA |
|  | Normal | 4 | 83 | 6 |
|  | Obese | 14 | 209 | 9 |
|  | Overweight | 7 | 115 | 7 |
|  | Underweight | NA | 4 | NA |
| Course of disorder continuous chronic vs relapsing-remitting | Continuous chronic | 9 | 99 | 6 |
|  | Relapsing-remitting | 14 | 285 | 14 |
|  | NA | 2 | 30 | 2 |
| Alcohol / drug abuse within 1 year of onset | No | 11 | 191 | 8 |
|  | Yes | 14 | 223 | 14 |
| Alcohol abuse / dependence with psychopathology | No | 10 | 220 | 5 |
|  | Yes | 15 | 194 | 17 |
| Cannabis abuse / dependence with psychopathology | No | 11 | 205 | 9 |
|  | Yes | 14 | 209 | 13 |
| Coarse brain disease prior to onset | No | 24 | 381 | 21 |
|  | Yes | 1 | 33 | 1 |
| Lifetime diagnosis of alcohol abuse / dependence | No | 9 | 198 | 3 |
|  | Yes | 16 | 216 | 19 |
| Lifetime diagnosis of cannabis abuse / dependence | No | 11 | 194 | 4 |
|  | Yes | 14 | 220 | 18 |
| Lifetime diagnosis of other abuse / dependence | No | 14 | 277 | 12 |
|  | Yes | 11 | 137 | 10 |
| Other abuse / dependence with psychopathology | No | 18 | 292 | 13 |
|  | Yes | 7 | 122 | 9 |
| DSCT - median dichotomy | High | 11 | 205 | 11 |
|  | Low | 13 | 189 | 11 |
|  | NA | 1 | 20 | NA |
| Calculated duration of illness - median dichotomy | High | 15 | 214 | 14 |
|  | Low | 10 | 199 | 8 |
|  | NA | NA | 1 | NA |
| Family history of heart disease | No | 20 | 269 | 12 |
|  | Yes | 5 | 130 | 10 |
|  | NA | NA | 15 | NA |
| Family history of obesity | No | 21 | 286 | 12 |
|  | Yes | 3 | 116 | 10 |
|  | NA | 1 | 12 | NA |
| Family history of psychiatric disorder other than schizophrenia | No | 16 | 217 | 7 |
|  | Yes | 9 | 197 | 15 |
| Family history of schizophrenia | Yes | 8 | 115 | 4 |
|  | No | 17 | 299 | 18 |
| Harmonized Metabolic syndrome | Yes | 14 | 215 | 10 |
|  | No | 9 | 154 | 10 |
|  | NA | 2 | 45 | 2 |
| Lifetime: any delusions | No | 3 | 65 | 6 |
|  | Yes | 22 | 349 | 16 |
| Lifetime: any depressive symptoms | No | 3 | 85 | 4 |
|  | Yes | 22 | 329 | 18 |
| Lifetime: any hallucinations | No | 4 | 86 | 3 |
|  | Yes | 21 | 328 | 19 |
| Lifetime: any subjective thought disorder | No | 6 | 224 | 13 |
|  | Yes | 19 | 190 | 9 |
| Lifetime: symptoms of mania - elevated or irritable mood | No | 12 | 258 | 13 |
|  | Yes | 13 | 156 | 9 |
| Medical history: Allergies | No | 20 | 328 | 17 |
|  | Yes | 5 | 86 | 5 |
| Medical history: Arthritis | No | 20 | 327 | 21 |
|  | Yes | 5 | 86 | 1 |
|  | NA | NA | 1 | NA |
| Medical history: Asthma | No | 21 | 279 | 15 |
|  | Yes | 4 | 135 | 7 |
| Mood stabiliser with lamotrigine | No | 21 | 300 | 15 |
|  | Yes | 4 | 114 | 7 |
| NART Fullscale IQ - median dichotomy | High | 12 | 198 | 5 |
|  | Low | 9 | 177 | 14 |
|  | NA | 4 | 39 | 3 |
| PSP dichotomy | Good functioning | 2 | 83 | 3 |
|  | Poor functioning | NA | 17 | 1 |
|  | NA | 23 | 314 | 18 |
| Sex | Female | 7 | 158 | 14 |
|  | Male | 18 | 256 | 8 |
| Typical antipsychotics | No | 23 | 341 | 19 |
|  | Yes | 2 | 73 | 3 |

**Table S5: Cohort demographics of psychopathological symptoms examined for associations with detected autoantibodies.**

| **Variable** | **Yes (n)** | **No (n)** | **No data (n)** |
| --- | --- | --- | --- |
| 3 or more negative Sx (Carpenter scale) | 245 | 216 | 0 |
| 2 or fewer negative Sx (Carpenter scale) | 216 | 245 | 0 |
| DSCT score above median (39) | 227 | 213 | 21 |
| DSCT score below median (39) | 213 | 227 | 21 |
| Lifetime: any delusions | 387 | 74 | 0 |
| Lifetime: any depressive symptoms | 369 | 92 | 0 |
| Lifetime: any hallucinations | 368 | 93 | 0 |
| Lifetime: symptoms of mania - elevated or irritable mood | 178 | 283 | 0 |
| Lifetime: any subjective thought disorder | 218 | 243 | 0 |
| NART score above median (99.6) | 215 | 200 | 46 |
| NART score below median (99.6) | 200 | 215 | 46 |
| Present state: any delusions | 179 | 282 | 0 |
| Present state: any depressive symptoms | 127 | 334 | 0 |
| Present state: any hallucinations | 159 | 302 | 0 |
| Present state: screen for mania | 32 | 429 | 0 |
| Present state: any subjective thought disorder | 96 | 365 | 0 |
| Past year or present state: any delusions | 255 | 206 | 0 |
| Past year or present state: any depressive symptoms | 240 | 221 | 0 |
| Past year or present state: any hallucinations | 248 | 213 | 0 |
| Past year or present state: symptoms of mania - elevated or irritable mood | 85 | 376 | 0 |
| Past year or present state: any subjective thought disorder | 138 | 323 | 0 |
| Dysphoria: lifetime | 366 | 95 | 0 |
| Dysphoria: present state | 124 | 337 | 0 |
| Dysphoria: past year | 223 | 238 | 0 |
| Loss of pleasure: lifetime | 328 | 133 | 0 |
| Loss of pleasure: present state | 104 | 357 | 0 |
| Loss of pleasure: past year | 204 | 257 | 0 |
| Suicidal ideation: lifetime | 324 | 137 | 0 |
| Suicidal ideation: present state | 65 | 396 | 0 |
| Suicidal ideation: past year | 137 | 324 | 0 |
| Diurnal variation: lifetime | 115 | 346 | 0 |
| Diurnal variation: present state | 37 | 424 | 0 |
| Diurnal variation: past year | 70 | 391 | 0 |
| Poor concentration: lifetime | 303 | 158 | 0 |
| Poor concentration: present state | 101 | 360 | 0 |
| Poor concentration: past year | 189 | 272 | 0 |
| Slowed activity: lifetime | 195 | 266 | 0 |
| Slowed activity: present state | 53 | 408 | 0 |
| Slowed activity: past year | 115 | 346 | 0 |
| Loss of energy: lifetime | 322 | 139 | 0 |
| Loss of energy, or tiredness: present state | 108 | 353 | 0 |
| Loss of energy: past year | 204 | 257 | 0 |
| Altered libido: lifetime | 219 | 242 | 0 |
| Altered libido: present state | 85 | 376 | 0 |
| Altered libido: past year | 142 | 319 | 0 |
| Poor appetite: lifetime | 238 | 223 | 0 |
| Poor appetite: present state | 64 | 397 | 0 |
| Poor appetite: past year | 132 | 329 | 0 |
| Increased appetite: lifetime | 151 | 310 | 0 |
| Increased appetite: present state | 65 | 396 | 0 |
| Increased appetite: past year | 103 | 358 | 0 |
| Initial insomnia: lifetime | 253 | 208 | 0 |
| Initial insomnia: present state | 86 | 375 | 0 |
| Initial insomnia: past year | 167 | 294 | 0 |
| Middle insomnia: lifetime | 228 | 233 | 0 |
| Middle insomnia: present state | 87 | 374 | 0 |
| Middle insomnia: past year | 149 | 312 | 0 |
| Early morning waking: lifetime | 176 | 285 | 0 |
| Early morning waking: present state | 71 | 390 | 0 |
| Early morning waking: past year | 113 | 348 | 0 |
| Excessive sleep: lifetime | 209 | 252 | 0 |
| Excessive sleep: present state | 69 | 392 | 0 |
| Excessive sleep: past year | 142 | 319 | 0 |
| Excessive self-reproach: lifetime | 204 | 257 | 0 |
| Excessive self-reproach: present state | 81 | 380 | 0 |
| Excessive self-reproach: past year | 127 | 334 | 0 |
| Delusions of guilt: lifetime | 57 | 404 | 0 |
| Delusions of guilt: present state | 19 | 442 | 0 |
| Delusions of guilt: past year | 34 | 427 | 0 |
| Delusions of poverty: lifetime | 58 | 403 | 0 |
| Delusions of poverty: present state | 22 | 439 | 0 |
| Delusions of poverty: past year | 36 | 425 | 0 |
| Nihilistic delusions: lifetime | 51 | 410 | 0 |
| Nihilistic delusions: present state | 14 | 447 | 0 |
| Nihilistic delusions: past year | 22 | 439 | 0 |
| Elevated mood: lifetime | 139 | 322 | 0 |
| Elevated mood: present state | 11 | 450 | 0 |
| Elevated mood: past year | 61 | 400 | 0 |
| Irritable mood: lifetime | 138 | 323 | 0 |
| Irritable mood: present state | 27 | 434 | 0 |
| Irritable mood: past year | 63 | 398 | 0 |
| Thoughts racing: lifetime | 155 | 306 | 0 |
| Thoughts racing: present state | 21 | 440 | 0 |
| Thoughts racing: past year | 70 | 391 | 0 |
| Distractibility: lifetime | 142 | 319 | 0 |
| Distractibility: present state | 27 | 434 | 0 |
| Distractibility: past year | 70 | 391 | 0 |
| Excessive activity: lifetime | 131 | 330 | 0 |
| Excessive activity: present state | 9 | 452 | 0 |
| Excessive activity: past year | 58 | 403 | 0 |
| Reduced need for sleep: lifetime | 138 | 323 | 0 |
| Reduced need for sleep: present state | 13 | 448 | 0 |
| Reduced need for sleep: past year | 62 | 399 | 0 |
| Reckless activity: lifetime | 124 | 337 | 0 |
| Reckless activity: present state | 9 | 452 | 0 |
| Reckless activity: past year | 51 | 410 | 0 |
| Increased sociability: lifetime | 117 | 344 | 0 |
| Increased sociability: present state | 7 | 454 | 0 |
| Increased sociability: past year | 55 | 406 | 0 |
| Increased self-esteem: lifetime | 101 | 360 | 0 |
| Increased self-esteem: present state | 8 | 453 | 0 |
| Increased self-esteem: past year | 45 | 416 | 0 |
| Hallucinations in any modality: lifetime | 363 | 98 | 0 |
| Hallucinations in any modality: present state | 153 | 308 | 0 |
| Hallucinations in any modality: past year | 240 | 221 | 0 |
| Other (non affective) auditory hallucinations: lifetime | 178 | 283 | 0 |
| Other (non affective) auditory hallucinations present state | 76 | 385 | 0 |
| Other (non affective) auditory hallucinations: past year | 108 | 353 | 0 |
| Accusatory voices: lifetime | 270 | 191 | 0 |
| Accusatory voices: present state | 113 | 348 | 0 |
| Accusatory voices: past year | 175 | 286 | 0 |
| Running commentary: lifetime | 157 | 304 | 0 |
| Running commentary: present state | 72 | 389 | 0 |
| Running commentary: past year | 109 | 352 | 0 |
| Third person auditory hallucination: lifetime | 129 | 332 | 0 |
| Third person auditory hallucination: present state | 57 | 404 | 0 |
| Third person auditory hallucination: past year | 91 | 370 | 0 |
| Thought insertion: lifetime | 139 | 322 | 0 |
| Thought insertion: present state | 52 | 409 | 0 |
| Thought insertion: past year | 85 | 376 | 0 |
| Thought broadcast: lifetime | 130 | 331 | 0 |
| Thought broadcast: present state | 47 | 414 | 0 |
| Thought broadcast: past year | 70 | 391 | 0 |
| Thought withdrawal: lifetime | 57 | 404 | 0 |
| Thought withdrawal: present state | 19 | 442 | 0 |
| Thought withdrawal: past year | 27 | 434 | 0 |
| Thought echo: lifetime | 98 | 363 | 0 |
| Thought echo: present state | 47 | 414 | 0 |
| Thought echo: past year | 63 | 398 | 0 |
| Primary delusions: lifetime | 156 | 305 | 0 |
| Primary delusions: present state | 42 | 419 | 0 |
| Primary delusions: past year | 82 | 379 | 0 |
| Delusions of passivity: lifetime | 74 | 387 | 0 |
| Delusions of passivity: present state | 22 | 439 | 0 |
| Delusions of passivity: past year | 35 | 426 | 0 |
| Persecutory delusions: lifetime | 336 | 125 | 0 |
| Persecutory delusions: present state | 118 | 343 | 0 |
| Persecutory delusions: past year | 189 | 272 | 0 |
| Delusions of influence: lifetime | 240 | 221 | 0 |
| Delusions of influence: present state | 71 | 390 | 0 |
| Delusions of influence: past year | 131 | 330 | 0 |
| Primary delusional perception: lifetime | 115 | 346 | 0 |
| Primary delusional perception: present state | 26 | 435 | 0 |
| Primary delusional perception: past year | 51 | 410 | 0 |
| Grandiose delusions: lifetime | 194 | 267 | 0 |
| Grandiose delusions: present state | 53 | 408 | 0 |
| Grandiose delusions: past year | 90 | 371 | 0 |
| Bizarre delusions: lifetime | 102 | 359 | 0 |
| Bizarre delusions: present state | 33 | 428 | 0 |
| Bizarre delusions: past year | 49 | 412 | 0 |
| Lack of insight: lifetime | 77 | 384 | 0 |
| Psychotic symptoms nonresponsive to antipsychotics: lifetime | 399 | 62 | 0 |
| Well organized delusions: lifetime | 179 | 282 | 0 |
| Widespread delusions: lifetime | 274 | 187 | 0 |
| Delusions & hallucinations last for one week: lifetime | 300 | 161 | 0 |
| Persecutory delusions & hallucinations: lifetime | 263 | 198 | 0 |
| Agitated activity | 67 | 394 | 0 |
| Catatonia | 18 | 443 | 0 |
| Bizarre behaviour | 44 | 417 | 0 |
| Restricted affect | 187 | 274 | 0 |
| Blunted affect | 49 | 412 | 0 |
| Inappropriate affect | 41 | 420 | 0 |
| Pressure of speech | 65 | 396 | 0 |
| Speech difficult to understand | 17 | 444 | 0 |
| Positive formal thought disorder | 35 | 426 | 0 |
| Incoherence of speech | 6 | 455 | 0 |
| Negative formal thought disorder | 72 | 389 | 0 |
| Restricted affect | 199 | 262 | 0 |
| Poverty of speech | 72 | 389 | 0 |
| Diminished sense of purpose | 200 | 261 | 0 |
| Worrying (worried a lot more about things than other people) (last 12 mths) | 291 | 163 | 7 |
| General rating of anxiety (attack of fear or panic) (last 12 mths) | 215 | 242 | 4 |
| General ratings of phobia (last 12 mths) | 174 | 282 | 5 |
| Anxiety: can't get breath & smothering feeling (last 12 mths) | 135 | 137 | 189 |
| Anxiety: heart pounding, missing beats, beating faster (last 12 mths) | 198 | 74 | 189 |
| Anxiety: dizzy light headed, faint, or unsteady (last 12 mths) | 138 | 133 | 190 |
| Anxiety: tingling, numbness in face or fingers (last 12 mths) | 87 | 185 | 189 |
| Anxiety: tightness, discomfort or pain in your chest (last 12 mths) | 124 | 148 | 189 |
| Anxiety: dry mouth (last 12 mths) | 103 | 167 | 191 |
| Anxiety: difficulty swallowing, or lump in your throat (last 12 mths) | 93 | 179 | 189 |
| Anxiety: sweating (last 12 mths) | 138 | 134 | 189 |
| Anxiety: trembling or shaking (last 12 mths) | 157 | 115 | 189 |
| Anxiety: hot or cold sweats or flushes (last 12 mths) | 104 | 168 | 189 |
| Anxiety: feeling of not really being there (last 12 mths) | 99 | 173 | 189 |
| Anxiety: churning stomach, nausea or butterflies (last 12 mths) | 150 | 122 | 189 |
| Anxiety: fear of dying (last 12 mths) | 82 | 190 | 189 |
| Anxiety: feeling of choking (last 12 mths) | 50 | 221 | 190 |
| Anxiety: fear of going crazy, losing emotional control or passing out (last 12 mths) | 130 | 141 | 190 |
| Anxiety: apprehension, jumpiness, or increased startle response (last 12 mths) | 147 | 125 | 189 |
| Anxiety: anxiety or panic symptoms: other (last 12 mths) | 32 | 238 | 191 |
| Social phobia (last 12 mths) | 182 | 272 | 7 |
| Avoidance of social situations (last 12 mths) | 153 | 27 | 281 |
| Obsessional checking & repeating (last 12 mths) | 103 | 352 | 6 |
| Obsessional actions associated with excessive orderliness (last 12 mths) | 55 | 400 | 6 |
| Obsessional actions associated with cleanliness (last 12 mths) | 35 | 420 | 6 |

**Table S6: Cohort demographics of variables examined in secondary analysis of individuals with symptom-specific autoantibodies.** The level NA refers to missing data.

| **Variable** | **Level** | **n (%)** |
| --- | --- | --- |
| Affective vs non-affective disorders (DSM-IV) | Affective | 191 (41) |
|  | Non-affective | 244 (53) |
|  | NA | 26 (6) |
| Affective vs non-affective disorders (ICD-10) | Affective | 168 (36) |
|  | Non-affective | 248 (54) |
|  | NA | 45 (10) |
| Age at interview | 18-34 | 204 (44) |
|  | 35-64 | 257 (56) |
| Antidepressants | No | 278 (60) |
|  | Yes | 183 (40) |
| Any Cardiovascular disease | No | 415 (90) |
|  | Yes | 46 (10) |
| Atypical antipsychotic: clozapine | No | 378 (82) |
|  | Yes | 83 (18) |
| Atypical antipsychotics | No | 124 (27) |
|  | Yes | 337 (73) |
| BMI -criteria from World Health Organisation | No measurment | 3 (1) |
|  | Normal | 93 (20) |
|  | Obese | 232 (50) |
|  | Overweight | 129 (28) |
|  | Underweight | 4 (1) |
| Course of disorder continuous chronic vs relapsing-remitting | Continuous chronic | 114 (25) |
|  | Relapsing-remitting | 313 (68) |
|  | NA | 34 (7) |
| Alcohol / drug abuse within 1 year of onset | No | 210 (46) |
|  | Yes | 251 (54) |
| Alcohol abuse / dependence with psychopathology | No | 235 (51) |
|  | Yes | 226 (49) |
| Cannabis abuse / dependence with psychopathology | No | 225 (49) |
|  | Yes | 236 (51) |
| Coarse brain disease prior to onset | No | 426 (92) |
|  | Yes | 35 (8) |
| Lifetime diagnosis of alcohol abuse / dependence | No | 210 (46) |
|  | Yes | 251 (54) |
| Lifetime diagnosis of cannabis abuse / dependence | No | 209 (45) |
|  | Yes | 252 (55) |
| Lifetime diagnosis of other abuse / dependence | No | 303 (66) |
|  | Yes | 158 (34) |
| Other abuse / dependence with psychopathology | No | 323 (70) |
|  | Yes | 138 (30) |
| Calculated duration of illness - median dichotomy | High | 243 (53) |
|  | Low | 217 (47) |
|  | NA | 1 (0) |
| Family history of heart disease | No | 301 (65) |
|  | Yes | 145 (31) |
|  | NA | 15 (3) |
| Family history of obesity | No | 319 (69) |
|  | Yes | 129 (28) |
|  | NA | 13 (3) |
| Family history of psychiatric disorder other than schizophrenia | No | 240 (52) |
|  | Yes | 221 (48) |
| Family history of schizophrenia | Yes | 127 (28) |
|  | No | 334 (72) |
| Harmonized Metabolic syndrome | Yes | 239 (52) |
|  | No | 173 (38) |
|  | NA | 49 (11) |
| Mood stabiliser with lamotrigine | No | 336 (73) |
|  | Yes | 125 (27) |
| PSP dichotomy | Good functioning | 88 (19) |
|  | Poor functioning | 18 (4) |
|  | NA | 355 (77) |
| Sex | Female | 179 (39) |
|  | Male | 282 (61) |
| Typical antipsychotics | No | 383 (83) |
|  | Yes | 78 (17) |

# References

1 Zandian, A. *et al.* Untargeted screening for novel autoantibodies with prognostic value in first-episode psychosis. *Transl Psychiatry* **7**, e1177, doi:10.1038/tp.2017.160 (2017).

2 Pin, E. *et al.* in *Cerebrospinal Fluid (CSF) Proteomics: Methods and Protocols* (eds Enrique Santamaría & Joaquín Fernández-Irigoyen) 303-318 (Springer New York, 2019).
